# A platform to design and optimise fluorogenic scFvs for detection of interleukin 33

**DOI:** 10.1039/d5sc09634k

**Published:** 2026-03-23

**Authors:** Abigail E. Reese, Utsa Karmakar, Margherita Restori, Marcela A. Hermoso, George M. Church, Erkin Kuru, Marc Vendrell

**Affiliations:** a Centre for Inflammation Research, The University of Edinburgh EH16 4UU Edinburgh UK; b IRR Chemistry Hub, Institute for Regeneration and Repair, The University of Edinburgh EH16 4UU Edinburgh UK marc.vendrell@ed.ac.uk; c Department of Gastroenterology and Hepatology, University Medical Centre Groningen, University of Groningen The Netherlands; d Department of Genetics, Harvard Medical School Boston MA USA; e Wyss Institute for Biologically Inspired Engineering, Harvard University Boston MA 02115 USA; f Externa Biosciences Cambridge MA 02142 USA erkin@externa.bio

## Abstract

Direct measurement of interleukin-33 (IL-33) in biological systems is critical for understanding its role in inflammatory diseases. In this work, we have developed a platform for the discovery and optimisation of fluorogenic biosensors that are built from scFv protein scaffolds. Our approach combined site-specific fluorophore labelling and deep-learning protein design to identify BS-7 as a biosensor for wash-free detection of human IL-33 in cell supernatants.

## Abstract 2

We developed a chemical platform combining protein labelling and fluorophore identification to accelerate the optimisation of fluorogenic scFvs.

## Graphical abstract

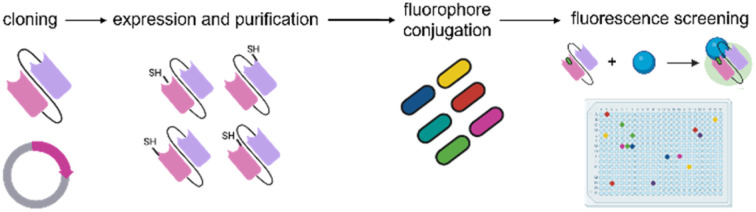

## Introduction

Interleukin-33 (IL-33) is a cytokine implicated in many inflammatory diseases, including asthma, atopic dermatitis, and inflammatory bowel diseases.^1^ Direct measurement of IL-33 is important in clinical diagnostics and for advancing our understanding of its role in immune regulation.^2^ Current detection methods, such as ELISA or immunohistochemistry, despite being well-established, are time-consuming and require wash steps that limit direct monitoring in biological systems.^3^ These limitations highlight the growing need for fluorogenic biosensors that can rapidly detect low-abundance interleukins like IL-33 under wash-free conditions and with minimal sample processing.

Fluorogenic biosensors are powerful tools for the visualization of cellular events, offering high sensitivity and specificity in biomolecular detection.^4^ Unlike always-on biosensors, fluorogenic constructs remain non-emissive until they bind their targets, enabling rapid detection without the need for amplification steps.^5^ However, the development of fluorogenic biosensors requires site-selective coupling of high-affinity recognition units to suitable OFF-to-ON reporters.^6^ For instance, our group has incorporated fluorescent amino acids into peptides for imaging apoptotic cells, pathogens and immune cells.^7^ Fluorogenic amino acids can be introduced into proteins by genetic code expansion but few structures are encodable due to limited incorporation efficiency.^8^

Antibody fragments such as nanobodies, diabodies, and single-chain variable fragments (scFvs) are compact high-affinity binders that are encoded as single polypeptide chains and can be readily expressed in *E. coli* bacterial cells.^9^ Among these, scFvs comprising the variable domains of heavy and light antibody chains exhibit robust folding efficiency and target recognition, making them excellent scaffolds for biosensor engineering.^10^ Previous studies have reported the use of scFvs as noncovalent fluorogen-activating structures for cell imaging; however, only a limited number of fluorogens and molecular constructs have been reported to date.^11^

**Fig. 1:**
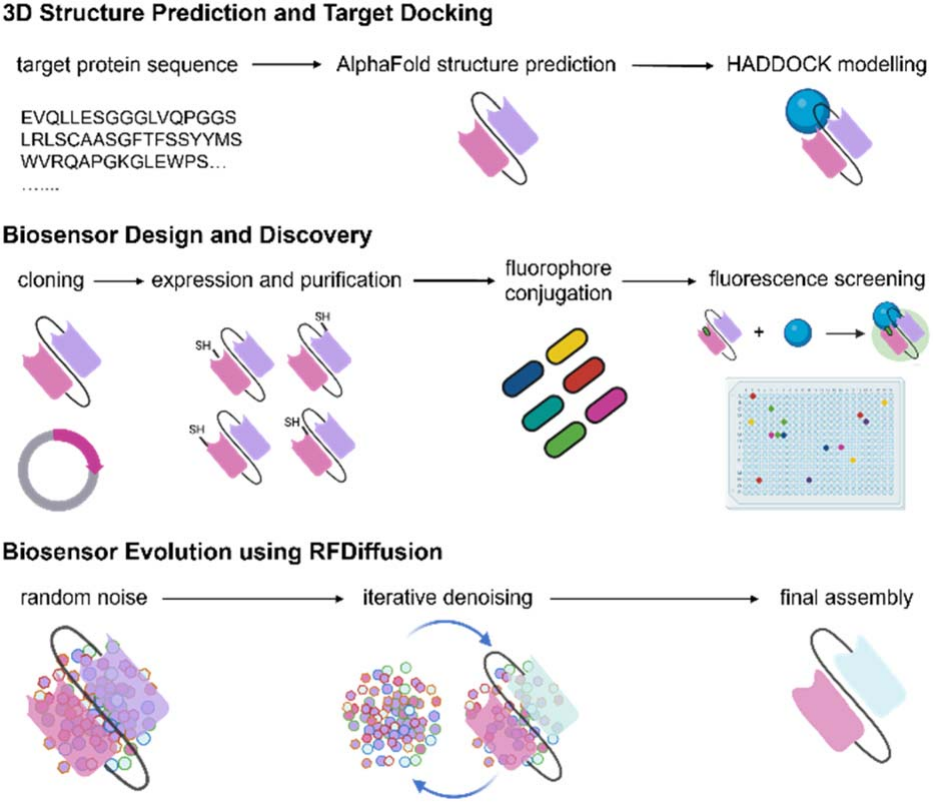
A platform for the design of optical biosensors based on fluorogenic scFvs. Schematic illustration of the workflow for the discovery and optimisation of fluorogenic scFv biosensors.

In this work, we designed a new platform for the discovery and optimisation of fluorogenic scFvs for detecting IL-33 (Fig. 1). First, we applied structure prediction to identify potential sites for fluorogen incorporation. Then, we prepared libraries of Cys knock-in mutants for site-selective labelling with diverse thiol-reactive fluorophores covering the entire visible spectrum. Systematic evaluation of all mutants-fluorogen combinations led to the identification of optimal residues and suitable fluorogenic dyes for scFv modification. Furthermore, we employed deep generative models, including RFDiffusion^12^ and ProteinMPNN,^13^ to refine initial scFv biosensors and enhance their fluorescence signal-to-noise ratios for detection of IL-33 in cell supernatants. This platform could be in principle applicable to the generation and evolution of fluorogenic scFvs targeting a wide range of biological targets.

## Results and discussion

### Structural prediction of fluorogenic biosensors for IL-33

C2_2E12 has been reported as a high-affinity scFv for IL-33 with minimal cross-reactivity against other IL-1-like cytokines.^14^ As a first step towards developing scFv-based fluorogenic biosensors for detection of IL-33, we sought to map the binding interface of C2_2E12 and IL-33 to guide targeted mutations within the scFv scaffold. This approach would enable to (1) site-specifically label Cys-containing C2_2E12 mutants with thiol-reactive fluorogens and (2) produce biosensors in *E. coli* in a scalable manner. Compared to full-length immunoglobulins, scFvs are stable scaffolds with straightforward chemical modification –due to the reduced number of disulphide bridges– as well as enhanced permeability and accessibility.^15^

To identify optimal residues in C2_2E12 that could tolerate the incorporation of fluorogenic dyes, we analysed the predicted complex between C2_2E12 and IL-33. In the absence of crystal structures, we employed AlphaFold2 to model the scFv tertiary structure.^16^ The prediction showed high confidence (predicted local distance difference test score > 90) and typical scFv architecture with well-formed VH and VL domains connected by a flexible linker.

**Fig. 2:**
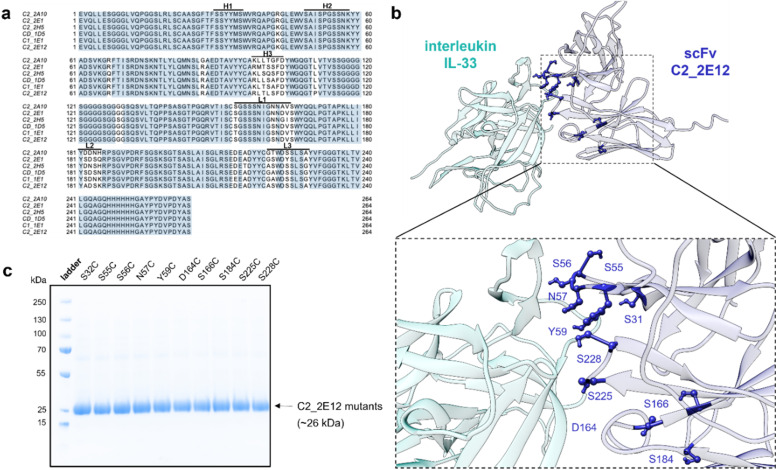
Design and expression of scFv mutants to bind human IL-33. (a) Sequence alignment of reported IL-33-binding scFvs. Sequences were manually entered to Jalview and conserved residues are highlighted in blue. (b) AlphaFold2 representation of the tertiary structure C2_2E12-IL-33. HADDOCK was used to predict the binding interface with IL-33 by biomolecular docking. (c) Representative gel from SDS-PAGE analysis of pure C2_2E12 mutants (Coomassie Blue).

C2_2E12 was originally isolated from a synthetic human scFv library with six complementarity-determining regions (CDRs).^17^ To identify potential binding regions, we aligned C2_2E12 to other IL-33-binding scFvs isolated from the same library. The CDRs H1 and H2 were highly conserved across these clones, suggesting their relevance for IL-33 recognition, whereas the CDRs H3, L1, L2, and L3 were more variable (Fig. 2a). We used regions H1 and H2 as restraints in HADDOCK^18^ to predict the complex between C2_2E12 andIL-33, and selected 10 residues at the binding interface for Cys knock-in mutations: Ser31, Ser55, Ser56, Asn57, Tyr59, Asp164, Ser166, Ser184, Ser225, and Ser228 (Fig. 2b). All 10 mutants were expressed as His-tagged proteins in SHuffle cells^19^ and purified by Immobilized Metal Affinity Chromatography (IMAC), yielding approximately 50 mg per litre of soluble protein with good purities as shown by SDS-PAGE analysis (Fig. 2c).

### The screening of C2_2E12-dye constructs identifies constructs with enhanced fluorescence upon IL-33 binding

**Fig. 3:**
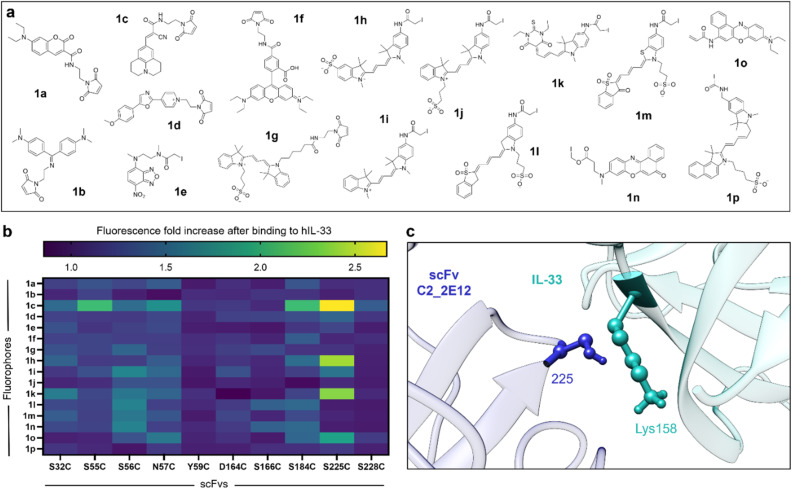
Screening of fluorogenic scFvs for human IL-33. (a) Chemical structures of thiol-reactive fluorophores coupled to Cys-containing C2_2E12 mutants: 1a, MDcC (430/470 nm); 1b, benzenamine maleimide (400/500 nm); 1c, CCVJ maleimide (435/500 nm); 1d, PyMPO maleimide (420/528 nm); 1e, IANBD (479/528 nm); 1f, rhodamine maleimide (530/560 nm); 1g, Cy3 maleimide (550/568 nm); 1h, sulfoCy3 iodoacetamide (550/568 nm); 1i, Cy3 iodoacetamide (550/568 nm); 1k, merocyanine iodoacetamide (585/610 nm); 1m, sulfonated merocyanine iodoacetamide (600/625 nm); 1n, Nile Red iodoacetamide (550/630 nm); 1o, Nile Blue acrylamide (635/674 nm); 1p, NIR 664 iodoacetamide (635/674 nm). (b) Heatmap summarising the fluorescence fold increases of all scFvs (50 µM) after incubation with hIL-33 (0.2 mg mL^−1^) in PBS (pH 7.5) at the respective excitation/emission wavelengths for each fluorophore. (c) Schematic illustration (using Chimera) of the binding interface between the original C2_2E12 (blue) and hIL-33 (cyan).

Following the expression of 10 Cys-containing C2_2E12 mutants (SI Fig. 1), we treated them with tris(2-carboxyethyl)phosphine (TCEP) to reduce the Cys and reacted them with 16 thiol-reactive fluorophores (1a–1p, Fig. 3a) using conventional bioconjugation protocols.^6*a*^ In order to cover a wide range of structural and optical diversity, we selected fluorophores based on different chemical scaffolds (*e.g.*, coumarin, cyanine, NBD, oxazone, rhodamine, cyanine, merocyanine) to produce a diverse library of potential fluorogenic biosensors. We confirmed the successful fluorophore conjugation by in-gel fluorescence SDS-PAGE analysis of a subset of fluorescent constructs (SI Fig. 2).

After fluorophore conjugation and purification by size exclusion (Zeba spin columns), we incubated all the fluorogenic C2_2E12 conjugates in PBS buffer (pH 7.5) in the presence or absence of human IL-33 (hIL-33) and measured the resulting fluorescence signals. Notably, we found that several positions within C2_2E12 (*e.g.*, Ser55, Ser56, Asn57, Ser184 and Ser225) led to enhanced fluorescence emission (Fig. 3b). Among them, the mutant S225C conjugated to the molecular rotor 9-(2-carboxy-2-cyanovinyl)julolidine (1c) showed the most prominent response after incubation with hIL-33 (*e.g.*, ∼3-fold fluorescence increase) as well as notable environmental sensitivity (SI Fig. 3). Other fluorophores with notable fluorogenicity included Cy3 (1h), merocyanine (1k) and Nile Red (1o) (Fig. 3b). Site-selective conjugation of the fluorogen 1c to Cys225 was confirmed by Synapt Q-TOF Protein Analysis mass spectrometry (SI Fig. 4), showing the addition of a single fluorophore with no labelling of the four native Cys.

Next, we evaluated the dose-dependent response of our hit biosensor (*i.e.*, C2_2E12 S225C with 1c coupled to Cys225, hereafter BS1) at increasing concentrations of hIL-33. We observed that BS1 showed fluorescence increase that was dependent on the concentration of hIL-33, with an apparent dissociation constant in the low micromolar range (SI Fig. 5), higher than the reported nanomolar affinity of the parent C2_2E12.^14^ Structural analysis using UCSF Chimera revealed that the residue Lys158 from hIL-33 extends into the binding interface close to the Cys225 (Fig. 3c). This proximity would (1) restrict the rotational freedom of 1c, with concomitant turn-on emission, and (2) potentially interfere with the binding to hIL-33, which would explain a reduction in binding affinity. Altogether, these results suggested that further chemical optimisation of the fluorogenic scFv BS1 could result in improved biosensors for hIL-33.

### Biosensor optimisation using deep-learning and *in silico* protein modelling

**Fig. 4:**
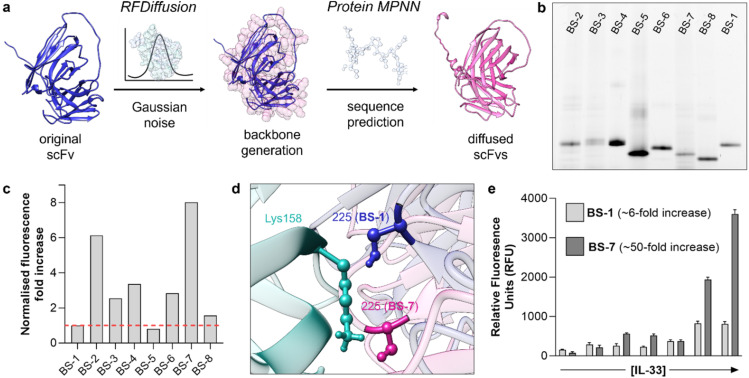
Biosensor optimisation using RFDiffusion and protein MPNN. (a) *In silico* workflow used to generate a library of diffused scFvs, combining RFDiffusion and ProteinMPNN for structure- and sequence-based design. (b) Representative in-gel fluorescence SDS-PAGE analysis of the new fluorogenic biosensors BS-1–BS-8. Excitation/emission: 435/500 nm. (c) Fluorescence fold increase of the new fluorogenic biosensors BS-2 to BS-7 (0.2 mg mL^−1^) after incubation with hIL-33 (0.2 mg mL^−1^) in PBS buffer (pH 7.5). Excitation/emission: 435/500 nm. (d) Schematic illustration of the binding interface formed by hIL-33 (cyan) and the two fluorogenic scFvs: BS-1 (blue) and BS-7 (pink). Structures visualized in UCSF Chimera. (e) Fluorescence responses of BS-1 and BS-7 (0.2 mg mL^−1^) after incubation in PBS buffer (pH 7.5) with increasing concentrations of hIL-33 (0.2, 1, 5, 25, 50, 100 µg mL^−1^). Excitation/emission: 435/500 nm.

Several computational tools have been described to generate *de novo* protein structures. In this work, we decided to employ RFDiffusion and ProteinMPNN to optimise the structure of BS1 as a biosensor for hIL-33 (Fig. 4a). RFDiffusion is a generative deep learning model that introduces Gaussian noise into protein structures and iteratively denoises them to produce refined backbones that retain key structural features with new configurations.^12^ ProteinMPNN complements this approach by predicting amino acid sequences that are likely to fold into a given backbone structure, leveraging a message-passing neural network trained on structural features.^5^

First, we applied RFDiffusion to the backbone of BS-1 while constraining its overall topology, allowing targeted exploration of alternative conformations at the binding interface with hIL-33. A constraint was applied in RFDiffusion to push Cys225 closer to the IL-33 interface. Afterwards, we employed ProteinMPNN to suggest protein sequences that could adopt the new backbone generated by RFDiffusion. From these sequences, we selected 7 constructs based on the lowest Rosetta energies. Sequence analysis confirmed that BS-2 to BS-5 retained close similarity to BS-1, including comparable VH and VL domains. In contrast, BS-6, BS-7, and BS-8 exhibited entirely novel sequences while preserving the relative positioning of residue 225 at the binding interface (SI Tables 1 and 2).

Next, we expressed all seven proteins in SHuffle cells and isolated them in amounts averaging 35 mg per litre of culture (SI Fig. 6). We also scaled up the synthesis of the maleimide-containing fluorophore 1c (Fig. 3a and experimental details in SI) to label all proteins and generate the corresponding biosensors BS2–BS8. As shown by SDS-PAGE gel analysis, all 7 biosensors were isolated in reasonable purities and migrated at the expected molecular weight (Fig. 4b and SI Fig. 7).

Finally, we evaluated the fluorogenic scFvs to identify the construct with the best sensitivity for hIL-33. With the exception of BS-5, all the new biosensors showed higher fluorescence increases than BS-1. Among them, BS-7 exhibited the highest fluorescence fold increase, and it was substantially brighter than BS-1 under the same experimental conditions (Fig. 4c). Further analysis of the binding interface using USCF Chimera confirmed the new positioning of residue 225, in closer proximity to Lys158 (Fig. 4d and SI Fig. 8). The identification of BS-7 demonstrates the ability of RFDiffusion to explore novel scaffolds with enhanced biosensing properties.

Direct comparison of BS-1 and BS-7 showed consistently brighter emission for the latter, with BS-1 showing a maximal 6-fold increase and BS-7 showing around 50-fold fluorescence increase under the same experimental conditions (Fig. 4e). Because fluorophore 1c behaves as a molecular rotor, this fluorescence enhancement is primarily driven by the restriction of rotational freedom at the binding interface and highlight BS-7 as a new fluorogenic scFv for hIL-33 and demonstrate the utility of combining RFDiffusion and ProteinMPNN for protein-based biosensor optimisation.

### BS-7 enables detection of secreted hIL-33 by human endothelial cells

hIL-33 is a cytokine expressed in the nuclear or perinuclear region of human umbilical vein endothelial cells (HUVEC) under homeostatic conditions.^20^ Although the release of hIL-33 from unstimulated HUVEC cells is minimal, the levels of IL-33 can substantially increase when HUVEC cells are incubated with stress agents like endosulfan,^21^ a known endocrine disruptor.

**Fig. 5:**
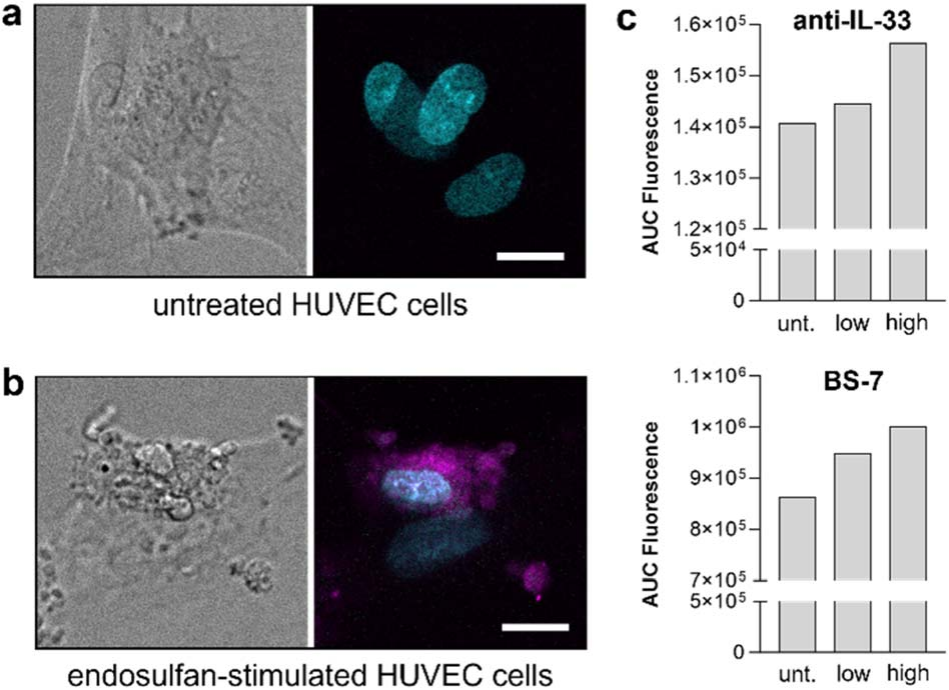
BS-7 detects endosulfan-triggered IL-33 secretion from HUVEC cells. (a and b) Confocal brightfield and fluorescence microscopy images of HUVEC cells without endosulfan (panel a) and after incubation with endosulfan (10 µg mL^−1^ for 24 h, panel b). HUVEC cells were counterstained with Hoechst (cyan) and AlexaFluor 647-conjugated anti-IL-33 antibody (magenta). Scale bars: 10 µm. (c) Representative fluorescence emission of HUVEC cells after incubation AlexaFluor 647-conjugated anti-IL-33 antibody (top, exc: 633 nm, em: 647 nm) and BS-7 (bottom, 100 nM, exc: 435 nm, em: 500 nm). Unt.: untreated, low: endosulfan 5 µg mL^−1^, high: endosulfan 10 µg mL^−1^.

First, we performed fluorescence microscopy experiments of HUVEC cells that had been cultured in the absence or presence of different concentrations of endosulfan (5–10 µg mL^−1^) for 24 hours. Endosulfan-treated and non-treated HUVEC cells were incubated with the same concentration of AlexaFluor 647-conjugated anti-IL-33 antibody and visualized under confocal fluorescence microscopy. As shown in Fig. 5a and b, endosulfan-treated HUVEC cells showed bright anti-IL33 fluorescence signals, whereas minimal staining was observed in HUVEC cells that had not been treated with endosulfan.

Finally, we performed HUVEC cell-based assays using the AlexaFluor 647-conjugated anti-IL-33 antibody or the BS-7 as fluorescent reporters of hIL-33. First, we cultured HUVEC cells with a low concentration (5 µg mL^−1^) or a high concentration (10 µg mL^−1^) of endosulfan for 24 h, followed by addition of anti-IL-33 antibody and BS-7 and subsequent measurements in a fluorescence plate reader. In both cases, we observed dose-dependent fluorescence readouts in endosulfan-treated HUVECs, in line with increased release of hIL-33 (Fig. 5c). Notably, fluorescence measurements of BS-7 were performed under wash-free conditions, confirming its fluorogenic nature and featuring the potential application of fluorogenic scFv-based constructs for the targeted detection of specific proteins.

## Conclusions

In summary, this work describes a chemical platform for the discovery and evolution of fluorogenic scFvs as optical biosensors, and proof-of-concept analysis for the detection of hIL-33. Building on C2_2E12 as a reported scFv for IL-33, we prepared a combinatorial collection of Cys knock-in mutants labelled with diverse thiol-reactive fluorophores. The screening of the collection against IL-33 highlighted that the residue 225 in C2_2E12 was close to the binding interface with IL-33 and suitable for fluorophore labelling. Moreover, the molecular rotor 1c showed the highest signal-to-background readouts among 12 fluorophores covering different fluorescent scaffolds and excitation and emission wavelengths. Building on the identification of BS-1 as a hit biosensor for IL-33, we employed RFDiffusion and ProteinMPNN to generate 7 additional constructs retaining both the Cys mutation in position 225 and the fluorophore 1c but including alternative protein domains. From this second collection, we identified BS-7 as a fluorogenic scFv with 50-fold fluorescence increase after incubation with IL-33. Furthermore, we employed BS-7 in cell-based assays with HUVEC cells to detect endosulfan-triggered secretion of hIL-33. We envisage that the combination of site-selective protein labelling and fluorophore identification will accelerate the production and optimisation of fluorogenic scFvs for different biological targets.

## Author contributions

A. E. R., U. K., M. R. and E. K. performed lab experiments. M. A. H. provided materials for biological testing. G. M. C. obtained funding for the study. A. E. R. and E. K. carried out computational modelling and data analysis. A. E. R., E. K. and M. V. prepared the initial drafts of the manuscript. M. V. conceived the study, obtained funding and wrote the final version of the manuscript with input from all authors.

## Conflicts of interest

G. M. C. and E. K. are co-founders of the company Externa Biosciences. The other co-authors declare no conflicts of interest.

## Supplementary Material

SC-OLF-D5SC09634K-s001

## Data Availability

The data supporting this article have been included as part of the supplementary information (SI). Supplementary information is available. See DOI: https://doi.org/10.1039/d5sc09634k.

## References

[cit1] Altman M. C., Lai Y., Nolin J. D., Long S., Chen C. C., Piliponsky A. M., Altemeier W. A., Larmore M., Frevert C. W., Mulligan M. S., Ziegler S. F., Debley J. S., Peters M. C., Hallstrand T. S. (2019). J. Clin. Invest..

[cit2] Wechsler M. E. (2021). et al.. N. Engl. J. Med..

[cit3] Li X., Soler M., Özdemir C. I., Belushkin A., Yesilköy F., Altug H. (2017). Lab Chip.

[cit4] Kielland N., Vendrell M., Lavilla R., Chang Y. (2012). Chem. Commun..

[cit5] Monsorno K. (2023). et al.. Nat. Commun..

[cit6] Fernandez A., Thompson E., Pollard J. W., Kitamura T., Vendrell M. (2019). Angew. Chem., Int. Ed..

[cit7] Barth N. D. (2020). et al.. Nat. Commun..

[cit8] Cheng Z., Kuru E., Sachdeva A., Vendrell M. (2020). Nat. Rev. Chem..

[cit9] de Aguiar R. B., de Almeida da Silva T., Costa B. A., Machado M. F. M., Yamada R. Y., Braggion C., Perez K. R. P., Mori M. A. S., Oliveira V., de Moraes J. Z. (2021). Sci. Rep..

[cit10] Wilson E. D., Probst D., Hamasaki M., Oda M., Kochar V., Xu Q., Tanaka A., Suzuki H., Asano R., Sode K. (2025). RSC Adv..

[cit11] Özhalici-Ünal H., Pow C. L., Marks S. A., Jesper L. D., Silva G. L., Shank N. I., Jones E. W., Burnette III J. M., Berget P. B., Armitage B. A. (2008). J. Am. Chem. Soc..

[cit12] Watson J. L. (2023). et al.. Nature.

[cit13] Ingraham J. B., Barzilay R., Jaakkola T. S. (2022). Science.

[cit14] Park S. B., Kim S.-J., Cho S. W., Choi C. Y., Lee S. (2020). Int. J. Mol. Sci..

[cit15] Kussia G. G., Tessema T. S. (2024). J. Immunol. Res..

[cit16] Gao M., Zhou H., Singh S. (2021). J. Chem. Inf. Model..

[cit17] Yang H. Y., Kang K. J., Chung J. E., Shim H. (2009). Mol. Cells.

[cit18] de Vries S. J., van Dijk M., Bonvin A. M. J. J. (2010). Nat. Protoc..

[cit19] Lobstein J., Emrich C. A., Jeans T., Faulkner M., Riggs G., Berkmen B. (2012). Microb. Cell Fact..

[cit20] Küchler A. M., Pollheimer J., Balogh J., Sponheim J., Manley L., Sørensen D. R., De Angelis P. M., Scott H., Haraldsen G. (2008). Am. J. Pathol..

[cit21] Zhang Z., Wei J., Ren L., Zhang J., Yang M., Jing L., Wang J., Sun Z., Zhou X. (2017). Environ. Sci. Pollut. Res. Int..

