# Supplementary material for: A platform to design and optimise fluorogenic scFvs for detection of interleukin 33
Source: Chem Sci. 2026 Mar 23;17(19):9501–6. doi: 10.1039/d5sc09634k (PMC13037505; doi:10.1039/d5sc09634k)
Supplement: SC-017-D5SC09634K-s001 [file SC-017-D5SC09634K-s001.pdf]

Supplementary Information (SI) for Chemical Science.
This journal is © The Royal Society of Chemistry 2026

Electronic Supporting Information

A Platform to Design and Optimise Fluorogenic scFvs for Detection of Interleukin 33

Table of Contents

1. Materials and Methods

2. Supplementary Figures

3. Supplementary Tables

4. Supplementary References

1. Materials and Methods

Commercial fluorophores employed in this study. Thiol-reactive fluorophores were purchased from commercial suppliers and used as received. Their chemical structures are included in Figure 3.

Compound	Chemical name	Supplier
**1a**	MDCpc	Sigma
**1d**	PyMPO maleimide	ThermoFisher
**1e**	IANBD	ThermoFisher
**1f**	Atto Rho3B maleimide	ATTO-Tec
**1g**	Cy3 maleimide	Lumiprobe
**1o**	Nile Blue acrylamide	Sigma
**1p**	NIR 664 iodoacetamide	Santa Cruz

Protein Sequence Alignment and Structural Modelling. The sequences of scFvs used in these experiments were obtained from a previous report.[1] Multiple sequence alignments of the scFv library were performed using Jalview (v2.11.2.5) to identify conserved regions and generate consensus sequences. Structural models of scFv sequences were predicted using the AlphaFold2 server (https://colab.research.google.com/github/sokrypton/ColabFold/blob/main/ AlphaFold2.ipynb). To predict the C2_2E12–hIL-33 binding interface, protein–protein docking was performed using HADDOCK 2.4 (https://wenmr.science.uu.nl/haddock2.4/) with the crystal structure of IL-33 (PDB ID: 2KII) as a template. Restraints were applied to outline key residues at the binding interface: residues 149–158 of IL-33 and CDR-H1/H2 of C2_2E12. Structural analysis of the resulting interface was performed using UCSF Chimera (https://www.cgl.ucsf.edu/chimera), and optimal residues for Cys substitution were selected for site-specific labelling.

Plasmid Cloning. Backbones for pET-28_TEV were amplified by PCR using Q5® High-Fidelity 2X Master Mix (NEB) with primers designed according to the manufacturer's instructions. Linearized backbones and eBlocks encoding protein sequences (IDT, 10 ng μL^{-1}) were assembled using Gibson Assembly® Master Mix (NEB) by incubation at 50 °C for 30 min. The assembly mix was diluted 1:3 with water and transformed into 75 µL ElectroMAX™ DH10B cells (ThermoFisher) by electroporation (1.80 kV, 1 pulse) using a Gene Pulser Xcell System (BioRad). Cells were immediately recovered in 500 µL SOC medium (NEB) and plated onto LB agar containing 50 µg/mL antibiotic. Single colonies were isolated from neat, 10×, and 50× dilutions. Constructs were verified by Sanger sequencing (Genewiz) and mini-prepped using either the QIAprep Spin Miniprep Kit (Qiagen) or Genewiz services. All constructs contained an N-terminal His tag and a TEV cleavage site.

Protein Expression. Plasmids encoding the scFvs and IL-33 variants were transformed into SHuffle® T7 or Rosetta (DE3) competent *E. coli* cells. Transformants were plated on LB agar containing 50 µg mL^{-1} kanamycin and incubated at 37 °C for 16 h. Individual colonies were inoculated into 80 mL MagicMedia (ThermoFisher) supplemented with 50 µg mL^{-1} kanamycin and grown at 30 °C with vigorous shaking for 16 h. Cells were harvested by centrifugation (5000 g, 30 min) and resuspended in BugBuster® Master Mix (4 mL g^{-1} cell pellet) for 45 min at r.t. Lysates were supplemented with 2 mL equilibration buffer (20 mM Tris-HCl, pH 8.3, 0.5 M NaCl, 5 mM imidazole) and centrifuged at 5,000 g for 15 min at 4 °C to remove insoluble material.

Protein Purification. Soluble scFv mutants were purified using HisPur™ Cobalt Resin (ThermoFisher). Clarified lysates were incubated with equilibrated resin (0.5 mL resin in 2 mL

buffer) for 35 min at r.t., centrifuged (700 × g, 2 min, 4 °C), washed twice with wash buffer (20 mM Tris–HCl, pH 8.3, 0.5 M NaCl, 20 mM imidazole), and eluted three times with 0.5 mL elution buffer (20 mM Tris–HCl, pH 8.3, 0.5 M NaCl, 200 mM imidazole). Protein concentrations were determined by measuring absorbance at 280 nm, and purities were qualitatively assessed by SDS–PAGE (Novex™ WedgeWell™ 10–20%, Invitrogen). Samples were prepared in Laemmli SDS sample buffer (ThermoFisher), run alongside a PageRuler™ Prestained Protein Ladder (ThermoFisher), and visualised by staining with GelCode™ Blue (ThermoFisher).

Fluorophore Conjugation. Proteins were diluted to 50 µM in PBS with 500 µM TCEP to reduce Cys and incubated overnight at 4 °C or 2 h at 25 °C. For labelling, 5 µL fluorophore (3.3 mM) was added to 45 µL protein in 96-well PCR plates and incubated overnight at 4 °C. Excess fluorophore was removed using Zeba spin desalting plates (7 kDa MWCO, ThermoFisher) pre-equilibrated in PBS. For His-tag removal, proteins were incubated with TEV protease (2 µL) and reaction buffer (10×, 10 µL) overnight at 4 °C, followed by incubation with HisPur™ Cobalt Resin (200 µL) to remove remaining His-tagged proteins. Purified fluorophore-conjugated scFv were exchanged to 1× PBS containing 10% glycerol and stored at 1 mg mL^{-1} at 4 °C for short term or at -80 °C for long term.

Fluorescence Screening and Dose-Response. For scFv screening, 2 µL fluorophore-conjugated scFv (0.2 mg mL^{-1}) were incubated with 2 µL hIL-33 (0.2 mg mL^{-1}) or PBS in low-volume 384-well black plates (Corning). Fluorescence was measured on a Biotek Synergy H1 plate reader at 25 °C and at the corresponding fluorophore excitation/emission wavelengths. Dose-response curves were obtained by serial dilution of commercial IL-33 (highest

concentration 1 mg mL^{-1}) with 2 μL biosensor (0.2 mg mL^{-1}). Background-subtracted fluorescence data were fitted using the Hill model in GraphPad Prism 9.

Biosensor Optimisation Using Deep-Learning and In Silico Protein Modelling. To optimise BS-1 as a biosensor for hIL-33, a computational workflow combining RFDiffusion and ProteinMPNN was employed. Structure generation was performed using the Google Colab notebook *RFDiffusion v1.1.1* (https://colab.research.google.com/github/sokrypton/ColabDesign/blob/v1.1.1/rf/examples/diffusion.ipynb). RFDiffusion was applied to the BS-1 backbone (predicted PDB structure from AlphaFold2) while maintaining overall topology to explore alternative conformations at the hIL-33 binding interface. A positional constraint was introduced to reposition Cys225 closer to the IL-33 interface. Backbone structures generated by RFDiffusion were subsequently processed with ProteinMPNN to design amino acid sequences predicted to adopt the new conformations. Seven constructs with the lowest Rosetta energies (**BS-2** to **BS-8**) were selected for experimental testing. The new amino acid sequences were further analysed using AlphaFold2 to confirm the preservation of the characteristic scFv fold, comprising well-formed VH and VL domains connected by a short flexible linker.

Fluorescence detection of secreted hIL-33 by human endothelial cells. HUVEC cells were cultured in endothelial cell growth medium (Merck, C-22011). 10,000 cells (100 μL per well) were seeded on an 18-well IBIDI chamber (for imaging) or on a 96-well plate (for plate reader assay) for 24 hours followed by endosulfan treatment (5 μg mL^{-1} and 10 μg mL^{-1}) for another 24 h. For confocal imaging, cells were stained with anti-IL-33 antibody (1:50) or BS-7 (500 nM) for 1 h on ice (counterstaining with Hoechst; 1: 5000). Images were acquired at Leica Stellaris FALCON

and analysed by LAS X software (3.7.6.25997). For fluorescence measurements in a plate reader, cells were stained with anti-IL-33 antibody (1: 50, secondary antibody AlexaFluor647, exc. 640 nm; em. 681 nm) or BS-7 (500 nM, exc. 435 nm; em. 550 ± 50 nm) and fluorescence measurement were recorded at r.t.

Scale-up Chemical Synthesis of Fluorophore 1c.

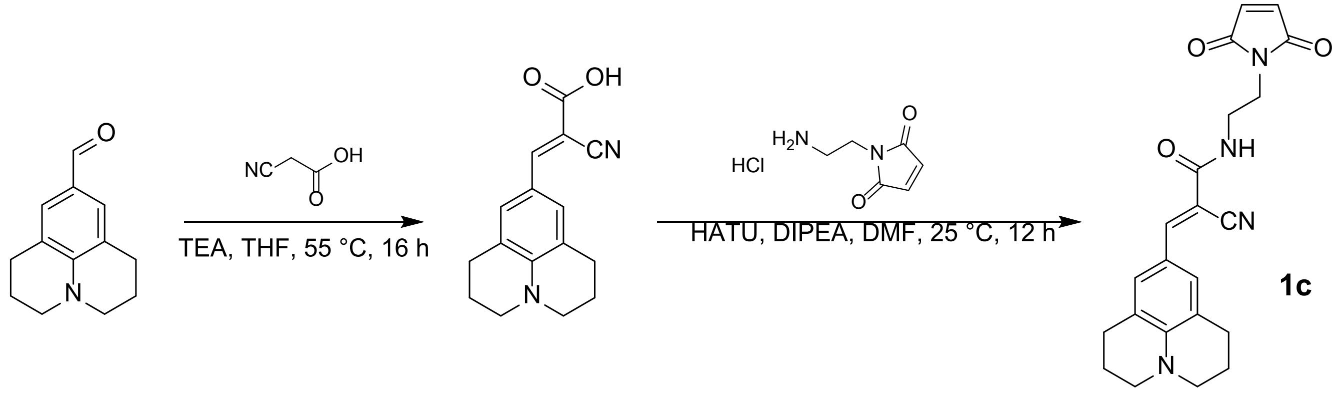

A 50 mL bottle was equipped with 1-azatricyclo[7.3.1.05,13]trideca-5(13),6,8-triene-7-carbaldehyde (2.5 g, 12.4 mmol, 1 eq.) and 2-cyanoacetic acid (1.6 g, 18.6 mmol, 1.5 eq.), the flask was sealed and purged with N_2 for 3 times. THF (15 mL) was added, and to the solution was added TEA (2.5 g, 24.8 mmol, 3.5 mL, 2 eq.) under N_2 atmosphere. The reaction mixture was stirred at 55°C for 16 h and concentrated under reduced pressure to give the crude product, which was purified by flash silica gel chromatography (DCM: MeOH, 0% to 100%) and concentrated under vacuum to give (*E*)-3-(1-azatricyclo[7.3.1.05,13]trideca-5,7,9(13)-trien-7-yl)-2-cyano-prop-2-enoic acid as a brown solid (2.1 g, 7.4 mmol, 60% yield, 95% purity).

ESI m/z: 269.0 $[M+H]^+$

^{1}**H NMR** (400 MHz, $CDCl_3$) δ: 7.93 (s, 1H), 7.48 (s, 2H), 3.29 (t, *J*=5.8 Hz, 4H), 2.73 (t, *J*=6.3 Hz, 4H), 1.94 (quin, *J*=6.0 Hz, 4H).

A solution of (*E*)-3-(1-azatricyclo[7.3.1.05,13]trideca-5,7,9(13)-trien-7-yl)-2-cyano-prop-2-enoic acid (200 mg, 745 μmol, 1 eq.) and HATU (425 mg, 1.1 mmol, 1.5 eq.) in DMF (2 mL) was stirred at 25 °C for 1 h, then 1-(2-aminoethyl)-1H-pyrrole-2, 5-dione; hydrogen chloride (158 mg, 895 μmol, 1.2 eq.) and DIPEA (192 mg, 1.5 mmol, 260 μL, 2 eq.) were added to the reaction mixture

and stirred at 25°C for 12 h. The reaction mixture was diluted with water (100 mL), extracted with ethyl acetate (3×100 mL) to give the combined organic layers, which were washed with brine (100 mL), dried over Na_2SO_4, filtered and concentrated under vacuum to give the crude product. The crude product was purified by silica flash column chromatography (PE: EA, 30% to 50% EA) and concentrated under reduced pressure to isolate compound **1c** as an orange solid (90 mg, 220 μmol, 30% yield, 96% purity).

ESI m/z: 391.1 $[M+H]^+$

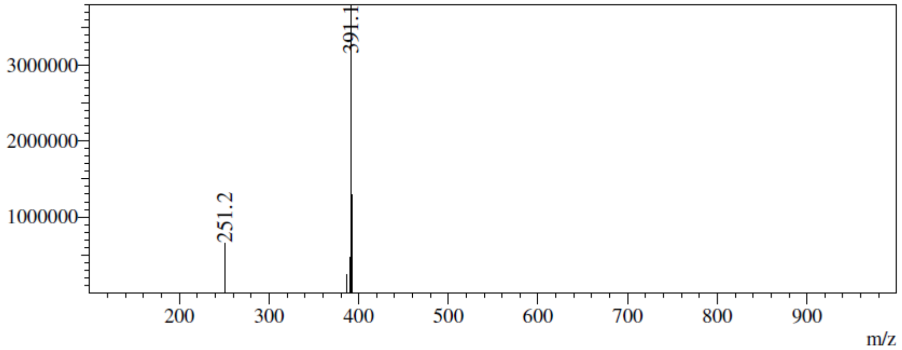

^{1}H NMR (400 MHz, DMSO-d6) δ: 8.09 (t, *J*=5.8 Hz, 1H), 7.74 (s, 1H), 7.41 (s, 2H), 7.02 (s, 2H), 3.54 (t, *J*=5.4 Hz, 2H), 3.32-3.27 (m, 6H), 2.68 (br t, *J*=6.1 Hz, 4H), 1.87 (quin, *J*=5.8 Hz, 4H).

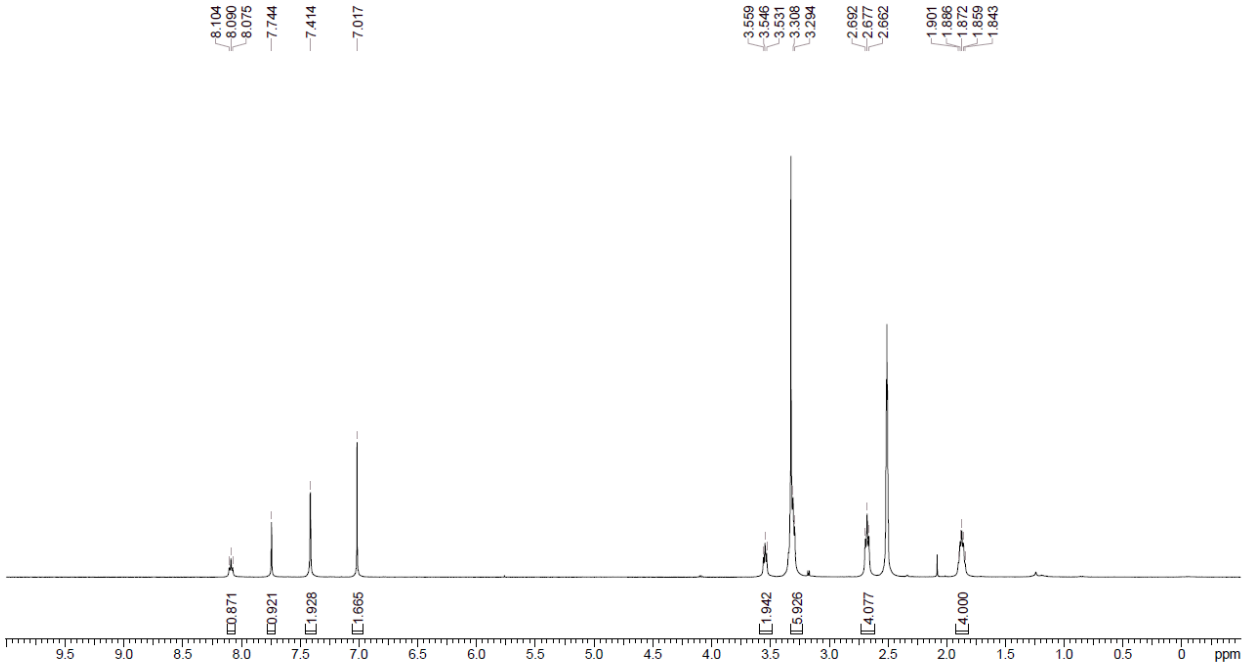

2. Supplementary Figures

Protein	Yield
S32C	60.3 mg L^{-1}
S55C	55.9 mg L^{-1}
S56C	37.8 mg L^{-1}
N57C	50.2 mg L^{-1}
Y59C	62.7 mg L^{-1}
D164C	43.1 mg L^{-1}
S166C	52.8 mg L^{-1}
S184C	39.9 mg L^{-1}
S225C	45.3 mg L^{-1}
S228C	42.6 mg L^{-1}

Figure S1. First protein library yields. Protein concentrations were measured on a DeNovix DS-11 spectrophotometer (280 nm) using the extinction coefficient of C2_2E12 (30,410 M^{-1} cm^{-1}).

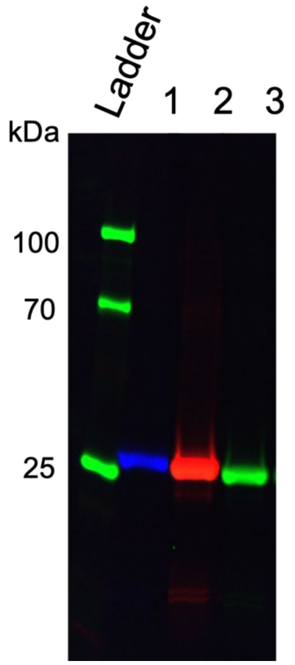

Figure S2. In-gel fluorescence SDS-PAGE analysis of S225C constructs conjugated to fluorophores 1c, 1h and 1k. Gels were imaged using suitable excitation/emission settings for each fluorophore: **1k** (lane 1): 585/610 nm, **1h** (lane 2): 550/568 nm, **1c** (lane 3): 435/500 nm.

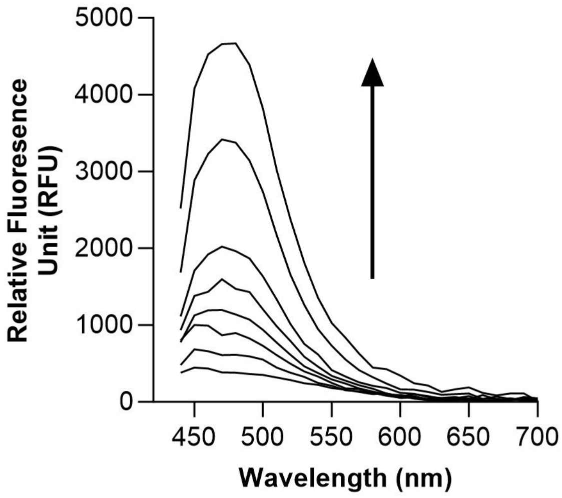

Figure S3. Fluorogenic response of fluorophore 1c. Compound **1c** (50 μM) was incubated with increasing concentrations of phosphatidylcholine liposomes (3.75 mg mL^{-1} to 0.004 mg mL^{-1} in two-fold serial dilutions) in PBS for 30 min. Excitation wavelength: 435 nm.

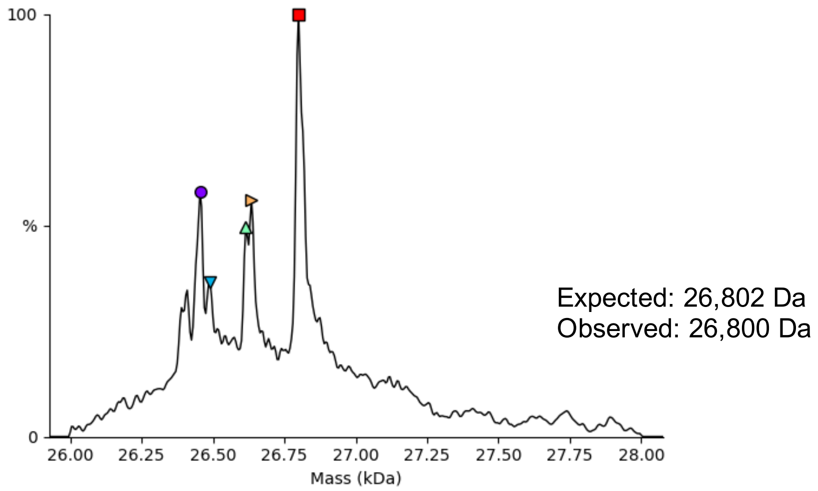

Figure S4. Mass spectrometry analysis of the biosensor BS-1. Synapt Q-TOF analysis (ESI) to confirm conjugation of fluorophore 1c to the protein.

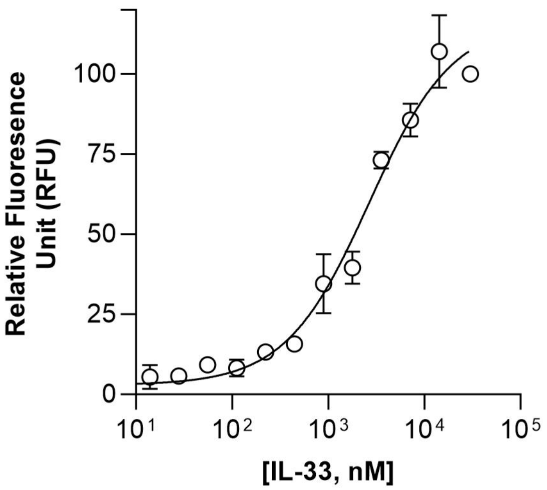

Figure S5. Dose-dependent fluorescence response of BS-1 with hIL-33. BS-1 (20 μM) was incubated with increasing concentrations of hIL-33 for 10 min in PBS. Values presented as means±SEM (n=3). Apparent K_D determined as of 3 μM. Excitation wavelength: 435 nm, emission wavelength: 500 nm.

Variant	ε ($M^{-1} cm^{-1}$)	Yield
**BS-2**	36,900	42.9 mg L^{-1}
**BS-3**	38,390	30.6 mg L^{-1}
**BS-4**	45,380	55.7 mg L^{-1}
**BS-5**	39,880	45.1 mg L^{-1}
**BS-6**	10,430	18.8 mg L^{-1}
**BS-7**	8,940	17.2 mg L^{-1}
**BS-8**	10,430	21.1 mg L^{-1}

Figure S6. Second protein library yields. Protein concentrations were measured on a Nanodrop One spectrophotometer (280 nm) using the corresponding extinction coefficient for every protein.

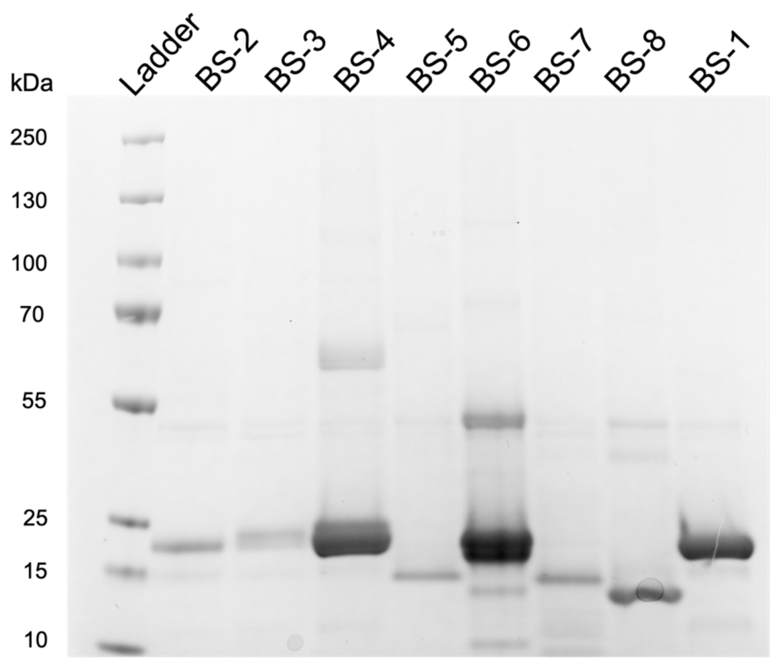

Figure S7. In-gel fluorescence SDS-PAGE analysis of the new fluorogenic biosensors BS-1-BS-8. Excitation wavelength: 435 nm, emission wavelength: 500 nm.

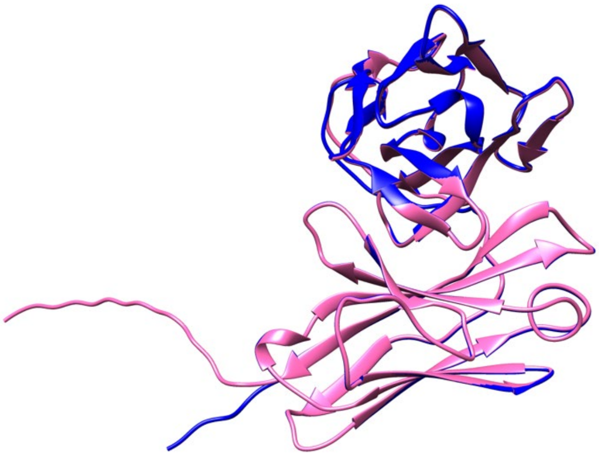

Figure S8. Structure comparison of the protein scaffolds in BS-1 (blue) and in BS-7 (pink).

Protein structures were predicted using AlphaFold2 and overlaid in UCSF Chimera.

3. Supplementary Tables

Table S1. Sequences of the gene constructs used in this study.

Construct	Sequence
**pET-28_TEV backbone**	AAGCTCAGGATCTGGTTCTGGTGGTGGTTCTGGTTAATAACGAAT<br>TCGAGCTCCGTCGACAAGCTTGCGGCCGCACTCGAGCACCACCA<br>CCACCACCACTGAGATCCGGCTGCTAACAAAGCCCGAAAGGAAG<br>CTGAGTTGGCTGCTGCCACCGCTGAGCAATAACTAGCATAACCCC<br>TTGGGGCCTCTAAACGGGTCTTGAGGGGTTTTTTGCTGAAAGGAG<br>GAACTATATCCGGATTGGCGAATGGGACGCGCCCTGTAGCGGCG<br>CATTAAGCGCGGCGGGTGTGGTGGTTACGCGCAGCGTGACCGCT<br>ACACTTGCCAGCGCCCTAGCGCCCGCTCCTTTCGCTTTCTTCCCT<br>TCCTTTCTCGCCACGTTCGCCGGCTTTCCCCGTCAAGCTCTAAAT<br>CGGGGGCTCCCTTTAGGGTTCCGATTTAGTGCTTTACGGCACCTC<br>GACCCCAAAAAACTTGATTAGGGTGATGGTTCACGTAGTGGGCCA<br>TCGCCCTGATAGACGGTTTTTCGCCCTTTGACGTTGGAGTCCACG<br>TTCTTTAATAGTGGACTCTTGTTCCAAACTGGAACAACACTCAACC<br>CTATCTCGGTCTATTCTTTTGATTTATAAGGGATTTTGCCGATTTCG<br>GCCTATTGGTTAAAAAATGAGCTGATTTAACAAAAATTTAACGCGA<br>ATTTTAACAAAATATTAACGTTTACAATTTCAGGTGGCACTTTTCGG<br>GGAAATGTGCGCGGAACCCCTATTTGTTTATTTTTCTAAATACATT<br>CAAATATGTATCCGCTCATGAATTAATTCTTAGAAAAACTCATCGA<br>GCATCAAATGAAACTGCAATTTATTCATATCAGGATTATCAATACCA<br>TATTTTTGAAAAAGCCGTTTCTGTAATGAAGGAGAAAACTCACCGA<br>GGCAGTTCCATAGGATGGCAAGATCCTGGTATCGGTCTGCGATTC<br>CGACTCGTCCAACATCAATACAACCTATTAATTTCCCCTCGTCAAA<br>AATAAGGTTATCAAGTGAGAAATCACCATGAGTGACGACTGAATC<br>CGGTGAGAATGGCAAAAGTTTATGCATTTCTTTCCAGACTTGTTCA<br>ACAGGCCAGCCATTACGCTCGTCATCAAAATCACTCGCATCAACC<br>AAACCGTTATTCATTCGTGATTGCGCCTGAGCGAGACGAAATACG<br>CGATCGCTGTTAAAAGGACAATTACAAACAGGAATCGAATGCAAC<br>CGGCGCAGGAACACTGCCAGCGCATCAACAATATTTTCACCTGAA<br>TCAGGATATTCTTCTAATACCTGGAATGCTGTTTTCCCGGGGATCG<br>CAGTGGTGAGTAACCATGCATCATCAGGAGTACGGATAAAATGCT<br>TGATGGTCGGAAGAGGCATAAATTCCGTCAGCCAGTTTAGTCTGA<br>CCATCTCATCTGTAACATCATTGGCAACGCTACCTTTGCCATGTTT<br>CAGAAACAACTCTGGCGCATCGGGCTTCCCATACAATCGATAGAT<br>TGTCGCACCTGATTGCCCGACATTATCGCGAGCCCATTTATACCC<br>ATATAAATCAGCATCCATGTTGGAATTTAATCGCGGCCTAGAGCAA<br>GACGTTTCCCGTTGAATATGGCTCATAACACCCCTTGTATTACTGT<br>TTATGTAAGCAGACAGTTTTATTGTTCATGACCAAAATCCCTTAAC<br>GTGAGTTTTCGTTCCACTGAGCGTCAGACCCCGTAGAAAAGATCA<br>AAGGATCTTCTTGAGATCCTTTTTTTCTGCGCGTAATCTGCTGCTT<br>GCAAACAAAAAAACCACCGCTACCAGCGGTGGTTTGTTTGCCGGA<br>TCAAGAGCTACCAACTCTTTTTCCGAAGGTAACTGGCTTCAGCAG

	AGCGCAGATACCAAATACTGTCCTTCTAGTGTAGCCGTAGTTAGG<br>CCACCACTTCAAGAACTCTGTAGCACCGCCTACATACCTCGCTCT<br>GCTAATCCTGTTACCAGTGGCTGCTGCCAGTGGCGATAAGTCGTG<br>TCTTACCGGGTTGGACTCAAGACGATAGTTACCGGATAAGGCGCA<br>GCGGTCGGGCTGAACGGGGGGTTCGTGCACACAGCCCAGCTTG<br>GAGCGAACGACCTACACCGAACTGAGATACCTACAGCGTGAGCTA<br>TGAGAAAGCGCCACGCTTCCCGAAGGGAGAAAGGCGGACAGGTA<br>TCCGGTAAGCGGCAGGGTCGGAACAGGAGAGCGCACGAGGGAG<br>CTTCCAGGGGGAAACGCCTGGTATCTTTATAGTCCTGTCGGGTTT<br>CGCCACCTCTGACTTGAGCGTCGATTTTTGTGATGCTCGTCAGGG<br>GGGCGGAGCCTATGGAAAAACGCCAGCAACGCGGCCTTTTTACG<br>GTTCCTGGCCTTTTGCTGGCCTTTTGCTCACATGTTCTTTCCTGCG<br>TTATCCCCTGATTCTGTGGATAACCGTATTACCGCCTTTGAGTGAG<br>CTGATACCGCTCGCCGCAGCCGAACGACCGAGCGCAGCGAGTCA<br>GTGAGCGAGGAAGCGGAAGAGCGCCTGATGCGGTATTTTCTCCT<br>TACGCATCTGTGCGGTATTTCACACCGCATATATGGTGCACTCTCA<br>GTACAATCTGCTCTGATGCCGCATAGTTAAGCCAGTATACACTCC<br>GCTATCGCTACGTGACTGGGTCATGGCTGCGCCCCGACACCCGC<br>CAACACCCGCTGACGCGCCCTGACGGGCTTGTCTGCTCCCGGCA<br>TCCGCTTACAGACAAGCTGTGACCGTCTCCGGGAGCTGCATGTGT<br>CAGAGGTTTTCACCGTCATCACCGAAACGCGCGAGGCAGCTGCG<br>GTAAAGCTCATCAGCGTGGTCGTGAAGCGATTCACAGATGTCTGC<br>CTGTTCATCCGCGTCCAGCTCGTTGAGTTTCTCCAGAAGCGTTAA<br>TGTCTGGCTTCTGATAAAGCGGGCCATGTTAAGGGCGGTTTTTTC<br>CTGTTTGGTCACTGATGCCTCCGTGTAAGGGGGATTTCTGTTCAT<br>GGGGGTAATGATACCGATGAAACGAGAGAGGATGCTCACGATAC<br>GGGTTACTGATGATGAACATGCCCGGTTACTGGAACGTTGTGAGG<br>GTAAACAACTGGCGGTATGGATGCGGCGGGACCAGAGAAAAATC<br>ACTCAGGGTCAATGCCAGCGCTTCGTTAATACAGATGTAGGTGTT<br>CCACAGGGTAGCCAGCAGCATCCTGCGATGCAGATCCGGAACAT<br>AATGGTGCAGGGCGCTGACTTCCGCGTTTCCAGACTTTACGAAAC<br>ACGGAAACCGAAGACCATTCATGTTGTTGCTCAGGTCGCAGACGT<br>TTTGCAGCAGCAGTCGCTTCACGTTCGCTCGCGTATCGGTGATTC<br>ATTCTGCTAACCAGTAAGGCAACCCCGCCAGCCTAGCCGGGTCCT<br>CAACGACAGGAGCACGATCATGCGCACCCGTGGGGCCGCCATGC<br>CGGCGATAATGGCCTGCTTCTCGCCGAAACGTTTGGTGGCGGGA<br>CCAGTGACGAAGGCTTGAGCGAGGGCGTGCAAGATTCCGAATAC<br>CGCAAGCGACAGGCCGATCATCGTCGCGCTCCAGCGAAAGCGGT<br>CCTCGCCGAAAATGACCCAGAGCGCTGCCGGCACCTGTCCTACG<br>AGTTGCATGATAAAGAAGACAGTCATAAGTGCGGCGACGATAGTC<br>ATGCCCCGCGCCCACCGGAAGGAGCTGACTGGGTTGAAGGCTCT<br>CAAGGGCATCGGTCGAGATCCCGGTGCCTAATGAGTGAGCTAAC<br>TTACATTAATTGCGTTGCGCTCACTGCCCGCTTTCCAGTCGGGAA<br>ACCTGTCGTGCCAGCTGCATTAATGAATCGGCCAACGCGCGGGG<br>AGAGGCGGTTTGCGTATTGGGCGCCAGGGTGGTTTTTCTTTTCAC<br>CAGTGAGACGGGCAACAGCTGATTGCCCTTCACCGCCTGGCCCT

	GAGAGAGTTGCAGCAAGCGGTCCACGCTGGTTTGCCCCAGCAGG CGAAAATCCTGTTTGATGGTGGTTAACGGCGGGATATAACATGAG CTGTCTTCGGTATCGTCGTATCCCACTACCGAGATATCCGCACCA ACGCGCAGCCCGGACTCGGTAATGGCGCGCATTGCGCCCAGCG CCATCTGATCGTTGGCAACCAGCATCGCAGTGGGAACGATGCCCT CATTCAGCATTTGCATGGTTTGTTGAAAACCGGACATGGCACTCC AGTCGCCTTCCCGTTCCGCTATCGGCTGAATTTGATTGCGAGTGA GATATTTATGCCAGCCAGCCAGACGCAGACGCGCCGAGACAGAA CTTAATGGGCCCGCTAACAGCGCGATTTGCTGGTGACCCAATGCG ACCAGATGCTCCACGCCCAGTCGCGTACCGTCTTCATGGGAGAAA ATAATACTGTTGATGGGTGTCTGGTCAGAGACATCAAGAAATAAC GCCGGAACATTAGTGCAGGCAGCTTCCACAGCAATGGCATCCTG GTCATCCAGCGGATAGTTAATGATCAGCCCACTGACGCGTTGCGC GAGAAGATTGTGCACCGCCGCTTTACAGGCTTCGACGCCGCTTC GTTCTACCATCGACACCACCACGCTGGCACCCAGTTGATCGGCG CGAGATTTAATCGCCGCGACAATTTGCGACGGCGCGTGCAGGGC CAGACTGGAGGTGGCAACGCCAATCAGCAACGACTGTTTGCCCG CCAGTTGTTGTGCCACGCGGTTGGGAATGTAATTCAGCTCCGCCA TCGCCGCTTCCACTTTTTCCCGCGTTTTCGCAGAAACGTGGCTGG CCTGGTTCACCACGCGGGAAACGGTCTGATAAGAGACACCGGCA TACTCTGCGACATCGTATAACGTTACTGGTTTCACATTCACCACCC TGAATTGACTCTCTTCCGGGCGCTATCATGCCATACCGCGAAAGG TTTTGCGCCATTCGATGGTGTCCGGGATCTCGACGCTCTCCCTTA TGCGACTCCTGCATTAGGAAGCAGCCCAGTAGTAGGTTGAGGCC GTTGAGCACCGCCGCCGCAAGGAATGGTGCATGCAAGGAGATGG CGCCCAACAGTCCCCCGGCCACGGGGCCTGCCACCATACCCACG CCGAAACAAGCGCTCATGAGCCCGAAGTGGCGAGCCCGATCTTC CCCATCGGTGATGTCGGCGATATAGGCGCCAGCAACCGCACCTG TGGCGCCGGTGATGCCGGCCACGATGCGTCCGGCGTAGAGGAT CGAGATCTCGATCCCGCGAAATTAATACGACTCACTATAGGGGAA TTGTGAGCGGATAACAATTCCCCTCTAGAAATAATTTTGTTTAACTT TAAGAAGGAGATATACCATGCATCACCATCATCATCATCATCACTC GTCA
**C2_2E12-D164C**	ATGCATCACCATCATCATCATCATCACTCGTCAGGCGAGAATCTTT ATTTTCAGAGCAGTGGTGAGGTCCAGTTGTTAGAATCGGGCGGG GGGTTGGTCCAACCAGGCGGTAGTTTACGTCTTTCCTGCGCGGC CTCCGGATTCACGTTTAGCTCATATTATATGTCATGGGTACGCCAG GCTCCAGGCAAGGGACTTGAGTGGGTATCCGCTATCAGCCCGGG CAGTTCGAATAAGTACTACGCAGACAGTGTCAAAGGCCGCTTTAC GATTAGTCGCGATAATTCGAAGAATACTTTATATCTTCAAATGAATT CACTGCGCGCGGAGGATACGGCGGTCTATTATTGTGCACGTCTG ACATTGTCGTTTGACTATTGGGGACAGGGGACACCTGTGACGGTC TCGTCTGGAGGCGGCGGTTCTGGCGGAGGCGGATCAGGTGGTG GAGGTAGCCAGTCAGTCTTAACTCAGCCTCCTTCCGCAAGCGGTA CCCCCGGTCAACGTGTAACTATTAGTTGTTCCGGCAGTTCTTCGA ACATCGGCTCGAATTGTGTCTCGTGGTACCAGCAATTACCTGGGA

	CGGCACCAAAGTTATTAATTTACGCAGATTCCAAACGTCCATCAGG TGTACCCGACCGTTTTTCCGGCTCAAAGTCGGGAACTTCCGCATC GCTGGCTATCTCGGGCTTGCGTTCGGAGGATGAAGCAGACTACTA TTGCGCATCGTGGGATTCCAGTTTATCGGCATACGTGTTCGGCGG GGGTACAAAGTTAACTGTTCTTGGTCAGGCAGGCCAGAGCTCAGG ATCTGGTTCTGGTGGTGGTTCTGGTTAA
**C2_2E12-S55C**	ATGCATCACCATCATCATCATCATCACTCGTCAGGCGAGAATCTTT ATTTTCAGAGCAGTGGTGAGGTCCAGTTGTTAGAATCGGGCGGG GGGTTGGTCCAACCAGGCGGTAGTTTACGTCTTTCCTGCGCGGC CTCCGGATTCACGTTTAGCTCATATTATATGTCATGGGTACGCCAG GCTCCAGGCAAGGGACTTGAGTGGGTATCCGCTATCAGCCCGGG CTGTTCGAATAAGTACTACGCAGACAGTGTCAAAGGCCGCTTTAC GATTAGTCGCGATAATTCGAAGAATACTTTATATCTTCAAATGAATT CACTGCGCGCGGAGGATACGGCGGTCTATTATTGTGCACGTCTG ACATTGTCGTTTGACTATTGGGGACAGGGGACACCTGTGACGGTC TCGTCTGGAGGCGGCGGTTCTGGCGGAGGCGGATCAGGTGGTG GAGGTAGCCAGTCAGTCTTAACTCAGCCTCCTTCCGCAAGCGGTA CCCCCGGTCAACGTGTAACTATTAGTTGTTCCGGCAGTTCTTCGA ACATCGGCTCGAATGATGTCTCGTGGTACCAGCAATTACCTGGGA CGGCACCAAAGTTATTAATTTACGCAGATTCCAAACGTCCATCAGG TGTACCCGACCGTTTTTCCGGCTCAAAGTCGGGAACTTCCGCATC GCTGGCTATCTCGGGCTTGCGTTCGGAGGATGAAGCAGACTACTA TTGCGCATCGTGGGATTCCAGTTTATCGGCATACGTGTTCGGCGG GGGTACAAAGTTAACTGTTCTTGGTCAGGCAGGCCAGAGCTCAGG ATCTGGTTCTGGTGGTGGTTCTGGTTAA
**C2_2E12-N57C**	ATGCATCACCATCATCATCATCATCACTCGTCAGGCGAGAATCTTT ATTTTCAGAGCAGTGGTGAGGTCCAGTTGTTAGAATCGGGCGGG GGGTTGGTCCAACCAGGCGGTAGTTTACGTCTTTCCTGCGCGGC CTCCGGATTCACGTTTAGCTCATATTATATGTCATGGGTACGCCAG GCTCCAGGCAAGGGACTTGAGTGGGTATCCGCTATCAGCCCGGG CAGTTCGTGTAAGTACTACGCAGACAGTGTCAAAGGCCGCTTTAC GATTAGTCGCGATAATTCGAAGAATACTTTATATCTTCAAATGAATT CACTGCGCGCGGAGGATACGGCGGTCTATTATTGTGCACGTCTG ACATTGTCGTTTGACTATTGGGGACAGGGGACACCTGTGACGGTC TCGTCTGGAGGCGGCGGTTCTGGCGGAGGCGGATCAGGTGGTG GAGGTAGCCAGTCAGTCTTAACTCAGCCTCCTTCCGCAAGCGGTA CCCCCGGTCAACGTGTAACTATTAGTTGTTCCGGCAGTTCTTCGA ACATCGGCTCGAATGATGTCTCGTGGTACCAGCAATTACCTGGGA CGGCACCAAAGTTATTAATTTACGCAGATTCCAAACGTCCATCAGG TGTACCCGACCGTTTTTCCGGCTCAAAGTCGGGAACTTCCGCATC GCTGGCTATCTCGGGCTTGCGTTCGGAGGATGAAGCAGACTACTA TTGCGCATCGTGGGATTCCAGTTTATCGGCATACGTGTTCGGCGG GGGTACAAAGTTAACTGTTCTTGGTCAGGCAGGCCAGAGCTCAGG ATCTGGTTCTGGTGGTGGTTCTGGTTAA
**C2_2E12-L99C**	ATGCATCACCATCATCATCATCATCACTCGTCAGGCGAGAATCTTT ATTTTCAGAGCAGTGGTGAGGTCCAGTTGTTAGAATCGGGCGGG

	GGGTTGGTCCAACCAGGCGGTAGTTTACGTCTTTCCTGCGCGGC<br>CTCCGGATTCACGTTTAGCTCATATTATATGTCATGGGTACGCCAG<br>GCTCCAGGCAAGGGACTTGAGTGGGTATCCGCTATCAGCCCGGG<br>CAGTTCGAATAAGTACTACGCAGACAGTGTCAAAGGCCGCTTTAC<br>GATTAGTCGCGATAATTCGAAGAATACTTTATATCTTCAAATGAATT<br>CACTGCGCGCGGAGGATACGGCGGTCTATTATTGTGCACGTTGTA<br>CATTGTCGTTTGACTATTGGGGACAGGGGACACCTGTGACGGTCT<br>CGTCTGGAGGCGGCGGTTCTGGCGGAGGCGGATCAGGTGGTGG<br>AGGTAGCCAGTCAGTCTTAACTCAGCCTCCTTCCGCAAGCGGTAC<br>CCCCGGTCAACGTGTAACTATTAGTTGTTCCGGCAGTTCTTCGAA<br>CATCGGCTCGAATGATGTCTCGTGGTACCAGCAATTACCTGGGAC<br>GGCACCAAAGTTATTAATTTACGCAGATTCCAAACGTCCATCAGGT<br>GTACCCGACCGTTTTTCCGGCTCAAAGTCGGGAACTTCCGCATCG<br>CTGGCTATCTCGGGCTTGCGTTCGGAGGATGAAGCAGACTACTAT<br>TGCGCATCGTGGGATTCCAGTTTATCGGCATACGTGTTCGGCGGG<br>GGTACAAAGTTAACTGTTCTTGGTCAGGCAGGCCAGAGCTCAGGA<br>TCTGGTTCTGGTGGTGGTTCTGGTTAA
**C2_2E12-S56C**	ATGCATCACCATCATCATCATCATCACTCGTCAGGCGAGAATCTTT<br>ATTTTCAGAGCAGTGGTGAGGTCCAGTTGTTAGAATCGGGCGGG<br>GGGTTGGTCCAACCAGGCGGTAGTTTACGTCTTTCCTGCGCGGC<br>CTCCGGATTCACGTTTAGCTCATATTATATGTCATGGGTACGCCAG<br>GCTCCAGGCAAGGGACTTGAGTGGGTATCCGCTATCAGCCCGGG<br>CAGTTGTAATAAGTACTACGCAGACAGTGTCAAAGGCCGCTTTAC<br>GATTAGTCGCGATAATTCGAAGAATACTTTATATCTTCAAATGAATT<br>CACTGCGCGCGGAGGATACGGCGGTCTATTATTGTGCACGTCTG<br>ACATTGTCGTTTGACTATTGGGGACAGGGGACACCTGTGACGGTC<br>TCGTCTGGAGGCGGCGGTTCTGGCGGAGGCGGATCAGGTGGTG<br>GAGGTAGCCAGTCAGTCTTAACTCAGCCTCCTTCCGCAAGCGGTA<br>CCCCCGGTCAACGTGTAACTATTAGTTGTTCCGGCAGTTCTTCGA<br>ACATCGGCTCGAATGATGTCTCGTGGTACCAGCAATTACCTGGGA<br>CGGCACCAAAGTTATTAATTTACGCAGATTCCAAACGTCCATCAGG<br>TGTACCCGACCGTTTTTCCGGCTCAAAGTCGGGAACTTCCGCATC<br>GCTGGCTATCTCGGGCTTGCGTTCGGAGGATGAAGCAGACTACTA<br>TTGCGCATCGTGGGATTCCAGTTTATCGGCATACGTGTTCGGCGG<br>GGGTACAAAGTTAACTGTTCTTGGTCAGGCAGGCCAGAGCTCAGG<br>ATCTGGTTCTGGTGGTGGTTCTGGTTAA
**C2_2E12-S116C**	ATGCATCACCATCATCATCATCATCACTCGTCAGGCGAGAATCTTT<br>ATTTTCAGAGCAGTGGTGAGGTCCAGTTGTTAGAATCGGGCGGG<br>GGGTTGGTCCAACCAGGCGGTAGTTTACGTCTTTCCTGCGCGGC<br>CTCCGGATTCACGTTTAGCTCATATTATATGTCATGGGTACGCCAG<br>GCTCCAGGCAAGGGACTTGAGTGGGTATCCGCTATCAGCCCGGG<br>CAGTTCGAATAAGTACTACGCAGACAGTGTCAAAGGCCGCTTTAC<br>GATTAGTCGCGATAATTCGAAGAATACTTTATATCTTCAAATGAATT<br>CACTGCGCGCGGAGGATACGGCGGTCTATTATTGTGCACGTCTG<br>ACATTGTCGTTTGACTATTGGGGACAGGGGACACCTGTGACGGTC<br>TCGTCTGGAGGCGGCGGTTCTGGCGGAGGCGGATCAGGTGGTG

	GAGGTAGCCAGTCAGTCTTAACTCAGCCTCCTTCCGCAAGCGGTA CCCCCGGTCAACGTGTAACTATTAGTTGTTCCGGCAGTTCTTCGA ACATCGGCTCGAATGATGTCTGTTGGTACCAGCAATTACCTGGGA CGGCACCAAAGTTATTAATTTACGCAGATTCCAAACGTCCATCAGG TGTACCCGACCGTTTTTCCGGCTCAAAGTCGGGAACTTCCGCATC GCTGGCTATCTCGGGCTTGCGTTCGGAGGATGAAGCAGACTACTA TTGCGCATCGTGGGATTCCAGTTTATCGGCATACGTGTTCGGCGG GGGTACAAAGTTAACTGTTCTTGGTCAGGCAGGCCAGAGCTCAGG ATCTGGTTCTGGTGGTGGTTCTGGTTAA
**C2_2E12-S228C**	ATGCATCACCATCATCATCATCATCACTCGTCAGGCGAGAATCTTT ATTTTCAGAGCAGTGGTGAGGTCCAGTTGTTAGAATCGGGCGGG GGGTTGGTCCAACCAGGCGGTAGTTTACGTCTTTCCTGCGCGGC CTCCGGATTCACGTTTAGCTCATATTATATGTCATGGGTACGCCAG GCTCCAGGCAAGGGACTTGAGTGGGTATCCGCTATCAGCCCGGG CAGTTCGAATAAGTACTACGCAGACAGTGTCAAAGGCCGCTTTAC GATTAGTCGCGATAATTCGAAGAATACTTTATATCTTCAAATGAATT CACTGCGCGCGGAGGATACGGCGGTCTATTATTGTGCACGTCTG ACATTGTCGTTTGACTATTGGGGACAGGGGACACCTGTGACGGTC TCGTCTGGAGGCGGCGGTTCTGGCGGAGGCGGATCAGGTGGTG GAGGTAGCCAGTCAGTCTTAACTCAGCCTCCTTCCGCAAGCGGTA CCCCCGGTCAACGTGTAACTATTAGTTGTTCCGGCAGTTCTTCGA ACATCGGCTCGAATGATGTCTCGTGGTACCAGCAATTACCTGGGA CGGCACCAAAGTTATTAATTTACGCAGATTCCAAACGTCCATCAGG TGTACCCGACCGTTTTTCCGGCTCAAAGTCGGGAACTTCCGCATC GCTGGCTATCTCGGGCTTGCGTTCGGAGGATGAAGCAGACTACTA TTGCGCATCGTGGGATTCCAGTTTATGTGCATACGTGTTCGGCGG GGGTACAAAGTTAACTGTTCTTGGTCAGGCAGGCCAGAGCTCAGG ATCTGGTTCTGGTGGTGGTTCTGGTTAA
**C2_2E12-Y33C**	ATGCATCACCATCATCATCATCATCACTCGTCAGGCGAGAATCTTT ATTTTCAGAGCAGTGGTGAGGTCCAGTTGTTAGAATCGGGCGGG GGGTTGGTCCAACCAGGCGGTAGTTTACGTCTTTCCTGCGCGGC CTCCGGATTCACGTTTAGCTCATATTGTATGTCATGGGTACGCCA GGCTCCAGGCAAGGGACTTGAGTGGGTATCCGCTATCAGCCCGG GCAGTTCGAATAAGTACTACGCAGACAGTGTCAAAGGCCGCTTTA CGATTAGTCGCGATAATTCGAAGAATACTTTATATCTTCAAATGAAT TCACTGCGCGCGGAGGATACGGCGGTCTATTATTGTGCACGTCTG ACATTGTCGTTTGACTATTGGGGACAGGGGACACCTGTGACGGTC TCGTCTGGAGGCGGCGGTTCTGGCGGAGGCGGATCAGGTGGTG GAGGTAGCCAGTCAGTCTTAACTCAGCCTCCTTCCGCAAGCGGTA CCCCCGGTCAACGTGTAACTATTAGTTGTTCCGGCAGTTCTTCGA ACATCGGCTCGAATGATGTCTCGTGGTACCAGCAATTACCTGGGA CGGCACCAAAGTTATTAATTTACGCAGATTCCAAACGTCCATCAGG TGTACCCGACCGTTTTTCCGGCTCAAAGTCGGGAACTTCCGCATC GCTGGCTATCTCGGGCTTGCGTTCGGAGGATGAAGCAGACTACTA TTGCGCATCGTGGGATTCCAGTTTATCGGCATACGTGTTCGGCGG

	GGGTACAAAGTTAACTGTTCTTGGTCAGGCAGGCCAGAGCTCAGG<br>ATCTGGTTCTGGTGGTGGTTCTGGTTAA
**C2_2E12-S31C**	ATGCATCACCATCATCATCATCATCACTCGTCAGGCGAGAATCTTT<br>ATTTTCAGAGCAGTGGTGAGGTCCAGTTGTTAGAATCGGGCGGG<br>GGGTTGGTCCAACCAGGCGGTAGTTTACGTCTTTCCTGCGCGGC<br>CTCCGGATTCACGTTTAGCTGTTATTATATGTCATGGGTACGCCAG<br>GCTCCAGGCAAGGGACTTGAGTGGGTATCCGCTATCAGCCCGGG<br>CAGTTCGAATAAGTACTACGCAGACAGTGTCAAAGGCCGCTTTAC<br>GATTAGTCGCGATAATTCGAAGAATACTTTATATCTTCAAATGAATT<br>CACTGCGCGCGGAGGATACGGCGGTCTATTATTGTGCACGTCTG<br>ACATTGTCGTTTGACTATTGGGGACAGGGGACACCTGTGACGGTC<br>TCGTCTGGAGGCGGCGGTTCTGGCGGAGGCGGATCAGGTGGTG<br>GAGGTAGCCAGTCAGTCTTAACTCAGCCTCCTTCCGCAAGCGGTA<br>CCCCCGGTCAACGTGTAACTATTAGTTGTTCCGGCAGTTCTTCGA<br>ACATCGGCTCGAATGATGTCTCGTGGTACCAGCAATTACCTGGGA<br>CGGCACCAAAGTTATTAATTTACGCAGATTCCAAACGTCCATCAGG<br>TGTACCCGACCGTTTTTCCGGCTCAAAGTCGGGAACTTCCGCATC<br>GCTGGCTATCTCGGGCTTGCGTTCGGAGGATGAAGCAGACTACTA<br>TTGCGCATCGTGGGATTCCAGTTTATCGGCATACGTGTTCGGCGG<br>GGGTACAAAGTTAACTGTTCTTGGTCAGGCAGGCCAGAGCTCAGG<br>ATCTGGTTCTGGTGGTGGTTCTGGTTAA
**C2_2E12-Y59C**	ATGCATCACCATCATCATCATCATCACTCGTCAGGCGAGAATCTTT<br>ATTTTCAGAGCAGTGGTGAGGTCCAGTTGTTAGAATCGGGCGGG<br>GGGTTGGTCCAACCAGGCGGTAGTTTACGTCTTTCCTGCGCGGC<br>CTCCGGATTCACGTTTAGCTCATATTATATGTCATGGGTACGCCAG<br>GCTCCAGGCAAGGGACTTGAGTGGGTATCCGCTATCAGCCCGGG<br>CAGTTCGAATAAGTGTTACGCAGACAGTGTCAAAGGCCGCTTTAC<br>GATTAGTCGCGATAATTCGAAGAATACTTTATATCTTCAAATGAATT<br>CACTGCGCGCGGAGGATACGGCGGTCTATTATTGTGCACGTCTG<br>ACATTGTCGTTTGACTATTGGGGACAGGGGACACCTGTGACGGTC<br>TCGTCTGGAGGCGGCGGTTCTGGCGGAGGCGGATCAGGTGGTG<br>GAGGTAGCCAGTCAGTCTTAACTCAGCCTCCTTCCGCAAGCGGTA<br>CCCCCGGTCAACGTGTAACTATTAGTTGTTCCGGCAGTTCTTCGA<br>ACATCGGCTCGAATGATGTCTCGTGGTACCAGCAATTACCTGGGA<br>CGGCACCAAAGTTATTAATTTACGCAGATTCCAAACGTCCATCAGG<br>TGTACCCGACCGTTTTTCCGGCTCAAAGTCGGGAACTTCCGCATC<br>GCTGGCTATCTCGGGCTTGCGTTCGGAGGATGAAGCAGACTACTA<br>TTGCGCATCGTGGGATTCCAGTTTATCGGCATACGTGTTCGGCGG<br>GGGTACAAAGTTAACTGTTCTTGGTCAGGCAGGCCAGAGCTCAGG<br>ATCTGGTTCTGGTGGTGGTTCTGGTTAA
**C2_2E12-S184C**	ATGCATCACCATCATCATCATCATCACTCGTCAGGCGAGAATCTTT<br>ATTTTCAGAGCAGTGGTGAGGTCCAGTTGTTAGAATCGGGCGGG<br>GGGTTGGTCCAACCAGGCGGTAGTTTACGTCTTTCCTGCGCGGC<br>CTCCGGATTCACGTTTAGCTCATATTATATGTCATGGGTACGCCAG<br>GCTCCAGGCAAGGGACTTGAGTGGGTATCCGCTATCAGCCCGGG<br>CAGTTCGAATAAGTACTACGCAGACAGTGTCAAAGGCCGCTTTAC

	GATTAGTCGCGATAATTCGAAGAATACTTTATATCTTCAAATGAATT<br>CACTGCGCGCGGAGGATACGGCGGTCTATTATTGTGCACGTCTG<br>ACATTGTCGTTTGACTATTGGGGACAGGGGACACCTGTGACGGTC<br>TCGTCTGGAGGCGGCGGTTCTGGCGGAGGCGGATCAGGTGGTG<br>GAGGTAGCCAGTCAGTCTTAACTCAGCCTCCTTCCGCAAGCGGTA<br>CCCCCGGTCAACGTGTAACTATTAGTTGTTCCGGCAGTTCTTCGA<br>ACATCGGCTCGAATGATGTCTCGTGGTACCAGCAATTACCTGGGA<br>CGGCACCAAAGTTATTAATTTACGCAGATTGTAAACGTCCATCAGG<br>TGTACCCGACCGTTTTTCCGGCTCAAAGTCGGGAACTTCCGCATC<br>GCTGGCTATCTCGGGCTTGCGTTCGGAGGATGAAGCAGACTACTA<br>TTGCGCATCGTGGGATTCCAGTTTATCGGCATACGTGTTCGGCGG<br>GGGTACAAAGTTAACTGTTCTTGGTCAGGCAGGCCAGAGCTCAGG<br>ATCTGGTTCTGGTGGTGGTTCTGGTTAA
**C2_2E12-S225C (BS-1)**	ATGCATCACCATCATCATCATCATCACTCGTCAGGCGAGAATCTTT<br>ATTTTCAGAGCAGTGGTGAGGTCCAGTTGTTAGAATCGGGCGGG<br>GGGTTGGTCCAACCAGGCGGTAGTTTACGTCTTTCCTGCGCGGC<br>CTCCGGATTCACGTTTAGCTCATATTATATGTCATGGGTACGCCAG<br>GCTCCAGGCAAGGGACTTGAGTGGGTATCCGCTATCAGCCCGGG<br>CAGTTCGAATAAGTACTACGCAGACAGTGTCAAAGGCCGCTTTAC<br>GATTAGTCGCGATAATTCGAAGAATACTTTATATCTTCAAATGAATT<br>CACTGCGCGCGGAGGATACGGCGGTCTATTATTGTGCACGTCTG<br>ACATTGTCGTTTGACTATTGGGGACAGGGGACACCTGTGACGGTC<br>TCGTCTGGAGGCGGCGGTTCTGGCGGAGGCGGATCAGGTGGTG<br>GAGGTAGCCAGTCAGTCTTAACTCAGCCTCCTTCCGCAAGCGGTA<br>CCCCCGGTCAACGTGTAACTATTAGTTGTTCCGGCAGTTCTTCGA<br>ACATCGGCTCGAATGATGTCTCGTGGTACCAGCAATTACCTGGGA<br>CGGCACCAAAGTTATTAATTTACGCAGATTCCAAACGTCCATCAGG<br>TGTACCCGACCGTTTTTCCGGCTCAAAGTCGGGAACTTCCGCATC<br>GCTGGCTATCTCGGGCTTGCGTTCGGAGGATGAAGCAGACTACTA<br>TTGCGCATCGTGGGATTGTAGTTTATCGGCATACGTGTTCGGCGG<br>GGGTACAAAGTTAACTGTTCTTGGTCAGGCAGGCCAGAGCTCAGG<br>ATCTGGTTCTGGTGGTGGTTCTGGTTAA
**BS-2**	ATGGAGGTGCAATTGCTTGAATCCGGGGGTGGGCTGGTACAGCC<br>CGGGGGGTCCTTGCGTTTAAGCTGCGCGGCATCGGGCTTTACGT<br>TTAACAGCGCGACGGCCTCTTGGGTCCGTCAGGCACCAGGCAAG<br>GGGCTTGAATGGGTTTCGGAAATCTCGTCATTCGGGGGTGTGGT<br>GAAGTACGCGGATTCTGTGAAGGGCCGCTTCACCATTTCGCGCG<br>ATAACTCCAAGAATACCCTTTACTTACAAATGAATAGTCTTCGTGC<br>TGAAGATACCGCAGTGTACTATTGCGCATTACATTTGAATAATACG<br>GTAACCTGGGGTCAGGGAACCCCTGTAACCGTATCATCAGGTGG<br>CGGAGGGTCAGGAGGAGGAGGCTCTGGCGGTGGAGGGTCACAA<br>AGCGTGCTTACGCAACCTCCTTCGGCATCAGGCACGCCCGGTCA<br>GCGCGTTACCATCAGTTGTAGCTTATCATCGGAGTACCCTCTGAC<br>ATCTGAGTTGAATTGGTATCAGCAACTTCCGGGAACGGCCCCCAA<br>ATTACTTATCTATAATAATATGCAGCGTGCTTCCGGAGTTCCTGAC<br>CGTTTTTCGGGATCTAAATCTGGGACTAGCGCCTCATTAGCAATCT

	CGGGATTACGTTCCGAGGACGAGGCGGATTATTACTGTCAGGCTC AAGTCTGTCTTGATCCTATGTACGTTTTTGGTGGGGGTACGAAGTT AACCGTTCTTGGACAGGCCGGTCAGTAA
**BS-3**	ATGGAAGTGCAGCTGCTTGAATCCGGTGGGGGCCTGGTCCAGCC GGGGGGGTCGCTTCGCTTATCATGTGCGGCGAGTGGATTTACTTT TAATGATGCCATCGCCCGTTGGGTCCGCCAAGCTCCTGGTAAGG GATTAGAGTGGGTGAGTACGATTGGACCAAATGGTGGCGTAGTGA CTTATGCAGATTCCGTAAAGGGACGTTTCACTATCAGCCGTGACA ATTCCAAGAACACGCTTTACCTGCAGATGAACTCCTTGCGTGCGG AGGATACCGCAGTCTACTATTGCGCCGCGGAGATCGACGGGCAA GTATATTGGGGACAAGGAACTCCGGTTACGGTATCGTCAGGTGG GGGCGGCTCTGGAGGGGGTGGATCAGGTGGTGGTGGGTCACAG AGTGTATTGACCCAACCGCCATCTGCGTCCGGTACGCCTGGACA GCGTGTCACTATCAGTTGTCAAATGCCTTCGACGCAGGCGGCTAA CTCAAAGGTGTATTGGTATCAGCAGCTTCCTGGTACGGCTCCCAA ATTACTTATTTACAACTCTACAAGCCTTCCGAGTGGAGTTCCCGAT CGTTTCTCCGGATCAAAATCGGGTACTAGCGCATCACTTGCCATTT CTGGGTTACGCTCTGAAGATGAAGCCGACTACTACTGCCAAGCCG AGGAGTGTGGTACGCTTGGTTATGTTTTCGGCGGAGGTACAAAGT TAACAGTCTTGGGTCAAGCCGGTCAGTAA
**BS-4**	ATGGAGGTTCAGTTACTGGAAAGCGGCGGTGGGTTGGTTCAGCC GGGGGGGTCGTTGCGCCTTAGCTGTGCGGCTTCGGGCTTCACAT TTTCGAGCTACCATTGGTCTTGGGTACGCCAGGCGCCGGGAAAG GGCCTGGAATGGGTGTCCTCAATCGACTCTGACTCGAAAAAAGTT CGCTATGCAGATAGTGTGAAGGGCCGCTTCACGATCTCCCGCGA CAATTCTAAGAACACACTTTACCTTCAGATGAACTCTTTACGTGCG GAAGACACAGCCGTATATTACTGTGCTGCCGAAGGCAATGACCAA GTGTATTGGGGTCAAGGCACACCGGTGACTGTTTCGTCGGGCGG AGGAGGTTCGGGCGGAGGGGGGTCAGGTGGTGGTGGGAGTCAA AGTGTCCTTACGCAACCGCCATCAGCGTCCGGCACGCCTGGGCA ACGTGTAACGATCTCATGTGACCTTTCTAGCATCAACGCTGGGGG GAGTAAACTGTACTGGTATCAGCAATTGCCGGGGACCGCCCCTAA ACTGTTAATTTACTCCAATAACAACCGCCCATCTGGGGTTCCGGA CCGTTTCTCAGGCTCGAAGAGCGGGACGTCTGCGAGCTTGGCAA TCTCTGGTTTACGTTCCGAGGACGAAGCGGATTACTATTGTCAAG CTGAAGATTGTGGGTCGGGCACTTATGTGTTTGGCGGTGGGACAA AGTTAACGGTCTTGGGGCAGGCCGGACAGTAA
**BS-5**	ATGGAGGTGCAGTTATTGGAATCCGGTGGCGGATTGGTCCAACCT GGTGGCAGCCTTCGCTTATCTTGTGCAGCTTCGGGGTTCACCTTT TCCAGCTACTACATGAGCTGGGTCCGTCAGGCACCGGGGAAAGG ACTGGAATGGGTCTCGGCAATTTCGCCTGGGTCCAGCAATAAATA TTACGCAGACAGCGTGAAGGGCCGTTTCACCATCTCACGTGACAA TTCAAAGAATACACTTTACCTTCAGATGAATTCTCTTCGCGCTGAA GACACAGCCGTTTATTACTGCGCACGCCTGACCTTGTCGTTTGAC TATTGGGGTCAAGGAACCCCTGTCACGGTTTCTTCTGGCGGAGGA GGGTCTGGCGGAGGTGGGTCCGGCGGTGGCGGCAGCCAATCTG

	TCTTAACTCAACCGCCGTCCGCCAGTGGTACCCCAGGTCAGCGT GTCACCATTTCGTGTTCAGGTTCAAGTTCGAATATTGGTAGCAATG ATGTATCTTGGTACCAGCAGCTGCCCGGTACTGCCCCGAAGTTGT TGATCTACGCGGACTCCAAACGTCCGTCGGGTGTACCCGATCGTT TCTCAGGCAGCAAATCGGGGACCTCAGCTAGTCTTGCGATCTCCG GGCTGCCAGCGGACGGCAACGCCCAGTTCTATTGTTTGGCACGC AGCTGTAAGCCTTACGGCTTTACCCCATCGTCTGCCACTACGCTG ACAGTGACCGGCCAGGCCGGTCAA
**BS-6**	ATGAGCATTACGACCGAAATTTCTGACTTGACAGTAACGGTTACG CCGGAATCTATTACGGTAAAGGCTACGCTTAAAAATGTCACTGAC GAAAGTGCAACCGTAGCGGTCTTACTGCAGACTAAGGACGGAGG GACCTCCACACTTTCTGTGGCGCGTTTAAATGAGAACATCAAAAA GGTTAACTCGGTCAGCAACCTGCCTGTTGAAACTACTGTCGACCT TAAGTCTGGCACGCTTACGGTAAAGATCCCCTATGTACCTAACTC GGAAGAGATTACGGTAACCGTCATGGTCTTCAAGGAGGGTAATTT TGAAATTCTTGCACTTGCTACAGCCACTGTCACGGTCGATTTGTCT GCCTTCACCGAAAAGCAAAAGAACTTTGAGCTGCCTGACAATACA ATCGACGATAAGACTGTGCTGAAGGTTGAAGAAGGTGAGACAGTT ACTTTTACTTACACGATCTCAGAAGAGTTAGAGAAAAATGGGTCTT ACTTTGTATTGGTAGAGTTCGAAGGCGGACCTACCAAGGCTATCT CAAGCAAAGAGAAAATCGTCGACGAAAAGGCCGGCGTTACTATCG AGGTCAAGCTTTCTAAGAAAACAATTACTATCACCATTTCGGGGTT GAAGGCCGGGGACAAACTTAAGATCACAGTATACTATTACGATTG TGAAACAGAAGAGTTCTATAAGCTGGCGACAGTTGAAATTGAAGT CGTTGAGTCAGAAGAAAAGTAA
**BS-7**	ATGGCAAAAATTACGTTGGTAGAGAAGGAACTGGGAAAGGAAGGC GAGGCTCATAAGTTAACCTTTGCAGTGTCTGACAAATCGCTGTTGA AATACACATTTCACGTCTTGGTCCAATTTGAAGGACAGCCTATTGC CTTAGCGCTGACGGTTGACCCAAGCGGTCGTTCCATTTACAACAG CGCAGAGGATATGAAATACGTGAAAATGACAGTGGATAACAAAAC TGGCACCCTTACGGTCGAACTGTACGGCGTACAGAAGGGGCAGA AGATTACCTTACGCGTTGTAGCTAGTGGAAAAGATGAAGTCGTCG TTTCAGAACCAGTAGTCATCGATACCAAGGACCCAAGCGAACCGC GCCAGAACATCGTAATCAACATGAAATTTTCTACGATCCAGCTTCC AAAAAAAGAAAATGTTGTCGTGAACGAGGGCGAGACAAAGACGGT CACATTCGAGCTGGGTGACCGCAAGCTGACCAAGGATCCAGTGA TTGGGTTAGTGGAATTCGAAGGGAAACCAGTAATCGCCATCCCCA CTGATGGCAAGTTATACATCGAAGAGACTGGGGTCGAGATCGAGA TCAAAACTGAGGAAAACAAGATCACGGTTACTATCAAGAACTTAAA GGTTGGAATGAAGATGACGTTGACGGTTGTAGCTCGCTCTTGTGA TGGTTATGTTGTCGTTGAAGTTGAGTTCGTAACGAATGAAAAGGA GGCGAAAGAAAAGGAATAA
**BS-8**	ATGAGTGTCGCCAAAGTGGAGGGAGTGAAAGAGGTGGAGGAAGG CGAGACTCTGACAATCACGATCAAATTCTCCGAAGAGTTAAAACC CGACGCCAACTTAATCTTGATCCTGGAGTTGCCCTCGGAGGGGC CGTACGCCGCAAATATGTCATTACGCGGAGACGGCAAGGTCACG

	TCCGTGTATTTAGAAGAAAGTGGGACGCTTATTAAAGTTGAACGCA<br>AAGGCAATACCCTTACCATCTCAATCAGTAATTTGGTAGGAGAAGA<br>TAGCGTAGAGGCTTATATCATCTTGATCGATGGGAGCGGGGAAAA<br>GGTGGTGGCGAGTGTAACGGTTGAGATTAAGGTAAAAACTAAGGA<br>GATCAAAAAATCGGCCAAAGAGCGCTTCAAAGATTTTGGAAAATTA<br>GAGGAGTTGCCCGAATTGAAGGTGAACGTGGGGGAATCAGTAAC<br>TATCACCGTAGAAATCAATGATAAGTTCGAAGATGAGCTGTTAGTA<br>GTGGTAATTGAGAAATATGAGGACGGCGAGCCCAAGGTCTATTTA<br>TTAAAAAAAGGTAAGAATAGCCCTGAGACTCCACCCTACTTGGAAA<br>TCGAGATGAAGAAAGGCAAGGTCACAGTGACATTAAAGGAACTTC<br>CCGAAATCGAAAAGTTAAAAATCTACGTACTGGGTGTAAACTGTGC<br>CGACTTTAGCACCCTGACCGGGAAAGCCTTGACTATCATTAATACT<br>TCACTGTTGGAGAAATAA

Table S2. Sequences of the proteins in this study.

Protein	Sequence
**C2_2E12-D164C**	MHHHHHHHHSSGENLYFQSSGEVQLLESGGGLVQPGGSLRLSCAASG FTFSSYYMSWVRQAPGKGLEWVSAISPGSSNKYYADSVKGRFTISRDNS KNTLYLQMNSLRAEDTAVYYCARLTLSFDYWGQGTPVTVSSGGGGSGG GGSGGGGSQSVLTQPPSASGTPGQRVTISCSGSSSNIGSNCVSWYQQL PGTAPKLLIYADSKRPSGVPDRFSGSKSGTSASLAISGLRSEDEADYYCA SWDSSLSAYVFGGGTKLTVLGQAGQSSGSGSGGGSG
**C2_2E12-S55C**	MHHHHHHHHSSGENLYFQSSGEVQLLESGGGLVQPGGSLRLSCAASG FTFSSYYMSWVRQAPGKGLEWVSAISPGCSNKYYADSVKGRFTISRDN SKNTLYLQMNSLRAEDTAVYYCARLTLSFDYWGQGTPVTVSSGGGGSG GGGSGGGGSQSVLTQPPSASGTPGQRVTISCSGSSSNIGSNDVSWYQ QLPGTAPKLLIYADSKRPSGVPDRFSGSKSGTSASLAISGLRSEDEADYY CASWDSSLSAYVFGGGTKLTVLGQAGQSSGSGSGGGSG
**C2_2E12-N57C**	MHHHHHHHHSSGENLYFQSSGEVQLLESGGGLVQPGGSLRLSCAASG FTFSSYYMSWVRQAPGKGLEWVSAISPGSSCKYYADSVKGRFTISRDNS KNTLYLQMNSLRAEDTAVYYCARLTLSFDYWGQGTPVTVSSGGGGSGG GGSGGGGSQSVLTQPPSASGTPGQRVTISCSGSSSNIGSNDVSWYQQL PGTAPKLLIYADSKRPSGVPDRFSGSKSGTSASLAISGLRSEDEADYYCA SWDSSLSAYVFGGGTKLTVLGQAGQSSGSGSGGGSG
**C2_2E12-L99C**	MHHHHHHHHSSGENLYFQSSGEVQLLESGGGLVQPGGSLRLSCAASG FTFSSYYMSWVRQAPGKGLEWVSAISPGSSNKYYADSVKGRFTISRDNS KNTLYLQMNSLRAEDTAVYYCARCTLSFDYWGQGTPVTVSSGGGGSG GGGSGGGGSQSVLTQPPSASGTPGQRVTISCSGSSSNIGSNDVSWYQ QLPGTAPKLLIYADSKRPSGVPDRFSGSKSGTSASLAISGLRSEDEADYY CASWDSSLSAYVFGGGTKLTVLGQAGQSSGSGSGGGSG
**C2_2E12-S56C**	MHHHHHHHHSSGENLYFQSSGEVQLLESGGGLVQPGGSLRLSCAASG FTFSSYYMSWVRQAPGKGLEWVSAISPGSCNKYYADSVKGRFTISRDN SKNTLYLQMNSLRAEDTAVYYCARLTLSFDYWGQGTPVTVSSGGGGSG GGGSGGGGSQSVLTQPPSASGTPGQRVTISCSGSSSNIGSNDVSWYQ QLPGTAPKLLIYADSKRPSGVPDRFSGSKSGTSASLAISGLRSEDEADYY CASWDSSLSAYVFGGGTKLTVLGQAGQSSGSGSGGGSG
**C2_2E12-S116C**	MHHHHHHHHSSGENLYFQSSGEVQLLESGGGLVQPGGSLRLSCAASG FTFSSYYMSWVRQAPGKGLEWVSAISPGSSNKYYADSVKGRFTISRDNS KNTLYLQMNSLRAEDTAVYYCARLTLSFDYWGQGTPVTVSSGGGGSGG GGSGGGGSQSVLTQPPSASGTPGQRVTISCSGSSSNIGSNDVCWYQQL PGTAPKLLIYADSKRPSGVPDRFSGSKSGTSASLAISGLRSEDEADYYCA SWDSSLSAYVFGGGTKLTVLGQAGQSSGSGSGGGSG
**C2_2E12-S228C**	MHHHHHHHHSSGENLYFQSSGEVQLLESGGGLVQPGGSLRLSCAASG FTFSSYYMSWVRQAPGKGLEWVSAISPGSSNKYYADSVKGRFTISRDNS KNTLYLQMNSLRAEDTAVYYCARLTLSFDYWGQGTPVTVSSGGGGSGG GGSGGGGSQSVLTQPPSASGTPGQRVTISCSGSSSNIGSNDVSWYQQL PGTAPKLLIYADSKRPSGVPDRFSGSKSGTSASLAISGLRSEDEADYYCA SWDSSLCAYVFGGGTKLTVLGQAGQSSGSGSGGGSG

**C2_2E12-Y33C**	MHHHHHHHHSSGENLYFQSSGEVQLLESGGGLVQPGGSLRLSCAASG<br>FTFSSYCMSWVRQAPGKGLEWVSAISPGSSNKYYADSVKGRFTISRDN<br>SKNTLYLQMNSLRAEDTAVYYCARLTLSFDYWGQGTPVTVSSGGGGSG<br>GGGSGGGGSQSVLTQPPSASGTPGQRVTISCSGSSSNIGSNDVSWYQ<br>QLPGTAPKLLIYADSKRPSGVPDRFSGSKSGTSASLAISGLRSEDEADYY<br>CASWDSSLSAYVFGGGTKLTVLGQAGQSSGSGSGGGSG
**C2_2E12-S31C**	MHHHHHHSSGENLYFQSSGEVQLLESGGGLVQPGGSLRLSCAASGFTF<br>SCYYMSWVRQAPGKGLEWVSAISPGSSNKYYADSVKGRFTISRDNSKN<br>TLYLQMNSLRAEDTAVYYCARLTLSFDYWGQGTPVTVSSGGGGSGGG<br>GSGGGGSQSVLTQPPSASGTPGQRVTISCSGSSSNIGSNDVSWYQQLP<br>GTAPKLLIYADSKRPSGVPDRFSGSKSGTSASLAISGLRSEDEADYYCAS<br>WDSSLSAYVFGGGTKLTVLGQAGQSSGSGSGGGSG
**C2_2E12-Y59C**	MHHHHHHHSSGENLYFQSSGEVQLLESGGGLVQPGGSLRLSCAASGFT<br>FSSYYMSWVRQAPGKGLEWVSAISPGSSNKCYADSVKGRFTISRDNSK<br>NTLYLQMNSLRAEDTAVYYCARLTLSFDYWGQGTPVTVSSGGGGSGGG<br>GSGGGGSQSVLTQPPSASGTPGQRVTISCSGSSSNIGSNDVSWYQQLP<br>GTAPKLLIYADSKRPSGVPDRFSGSKSGTSASLAISGLRSEDEADYYCAS<br>WDSSLSAYVFGGGTKLTVLGQAGQSSGSGSGGGSG
**C2_2E12-S184C**	MHHHHHHHHSSGENLYFQSSGEVQLLESGGGLVQPGGSLRLSCAASG<br>FTFSSYYMSWVRQAPGKGLEWVSAISPGSSNKYYADSVKGRFTISRDNS<br>KNTLYLQMNSLRAEDTAVYYCARLTLSFDYWGQGTPVTVSSGGGGSGG<br>GGSGGGGSQSVLTQPPSASGTPGQRVTISCSGSSSNIGSNDVSWYQQL<br>PGTAPKLLIYADCKRPSGVPDRFSGSKSGTSASLAISGLRSEDEADYYCA<br>SWDSSLSAYVFGGGTKLTVLGQAGQSSGSGSGGGSG
**C2_2E12-S225C (BS-1)**	MHHHHHHHHSSGENLYFQSSGEVQLLESGGGLVQPGGSLRLSCAASG<br>FTFSSYYMSWVRQAPGKGLEWVSAISPGSSNKYYADSVKGRFTISRDNS<br>KNTLYLQMNSLRAEDTAVYYCARLTLSFDYWGQGTPVTVSSGGGGSGG<br>GGSGGGGSQSVLTQPPSASGTPGQRVTISCSGSSSNIGSNDVSWYQQL<br>PGTAPKLLIYADSKRPSGVPDRFSGSKSGTSASLAISGLRSEDEADYYCA<br>SWDCSLSAYVFGGGTKLTVLGQAGQSSGSGSGGGSG
**BS-2**	MEVQLLESGGGLVQPGGSLRLSCAASGFTFNSATASWVRQAPGKGLE<br>WVSEISSFGGVVKYADSVKGRFTISRDNSKNTLYLQMNSLRAEDTAVYY<br>CALHLNNTVTWGQGTPVTVSSGGGGSGGGGSGGGGSQSVLTQPPSAS<br>GTPGQRVTISCSLSSEYPLTSELNWYQQLPGTAPKLLIYNNMQRASGVP<br>DRFSGSKSGTSASLAISGLRSEDEADYYCQAQVCLDPMYVFGGGTKLTV<br>LGQAGQ
**BS-3**	MEVQLLESGGGLVQPGGSLRLSCAASGFTFNDAIARWVRQAPGKGLEW<br>VSTIGPNGGVVTYADSVKGRFTISRDNSKNTLYLQMNSLRAEDTAVYYC<br>AAEIDGQVYWGQGTPVTVSSGGGGSGGGGSGGGGSQSVLTQPPSAS<br>GTPGQRVTISCQMPSTQAANSKVYWYQQLPGTAPKLLIYNSTSLPSGVP<br>DRFSGSKSGTSASLAISGLRSEDEADYYCQAEECGTLGYVFGGGTKLTV<br>LGQAGQ
**BS-4**	MEVQLLESGGGLVQPGGSLRLSCAASGFTFSSYHWSWVRQAPGKGLE<br>WVSSIDSDSKKVRYADSVKGRFTISRDNSKNTLYLQMNSLRAEDTAVYY<br>CAAEGNDQVYWGQGTPVTVSSGGGGSGGGGSGGGGSQSVLTQPPSA<br>SGTPGQRVTISCDLSSINAGGSKLYWYQQLPGTAPKLLIYSNNNRPSGVP

	DRFSGSKSGTSASLAISGLRSEDEADYYCQAEDCGSGTYVFGGGTKLTV LGQAGQ
**BS-5**	MEVQLLESGGGLVQPGGSLRLSCAASGFTFSSYYMSWVRQAPGKGLE WVSAISPGSSNKYYADSVKGRFTISRDNSKNTLYLQMNSLRAEDTAVYY CARLTLSFDYWGQGTPVTVSSGGGGSGGGGSGGGGSQSVLTQPPSAS GTPGQRVTISCSGSSSNIGSNDVSWYQQLPGTAPKLLIYADSKRPSGVP DRFSGSKSGTSASLAISGLPADGNAQFYCLARSCKPYGFTPSSATTLTVT GQAGQ
**BS-6**	MSITTEISDLTVTVTPESITVKATLKNVTDESATVAVLLQTKDGGTSTLSVA RLNENIKKVNSVSNLPVETTVDLKSGTLTVKIPYVPNSEEITVTVMVFKEG NFEILALATATVTVDLSAFTEKQKNFELPDNTIDDKTVLKVEEGETVTFTYT ISEELEKNGSYFVLVEFEGGPTKAISSKEKIVDEKAGVTIEVKLSKKTITITIS GLKAGDKLKITVYYYDCETEEFYKLATVEIEVVESEEK
**BS-7**	MAKITLVEKELGKEGEAHKLTFAVSDKSLLKYTFHVLVQFEGQPIALALTV DPSGRSIYNSAEDMKYVKMTVDNKTGTLTVELYGVQKGQKITLRVVASG KDEVVVSEPVVIDTKDPSEPRQNIVINMKFSTIQLPKKENVVVNEGETKTV TFELGDRKLTKDPVIGLVEFEGKPVIAIPTDGKLYIEETGVEIEIKTEENKIT VTIKNLKVGMKMTLTVVARSCDGYVVVEVEFVTNEKEAKEKE
**BS-8**	MSVAKVEGVKEVEEGETLTITIKFSEELKPDANLILILELPSEGPYAANMSL RGDGKVTSVYLEESGTLIKVERKGNTLTISISNLVGEDSVEAYIILIDGSGE KVVASVTVEIKVKTKEIKKSAKERFKDFGKLEELPELKVNVGESVTITVEIN DKFEDELLVVVIEKYEDGEPKVYLLKKGKNSPETPPYLEIEMKKGKVTVTL KELPEIEKLKIYVLGVNCADFSTLTGKALTIINTSLLEK

4. Supplementary References

[1] S. B. Park, S.-J. Kim, S. W. Cho, C. Y. Choi, S. Lee. *Int. J. Mol. Sci.* 2020, **21**, 6953.
